# Leveraging baseline transcriptional features and information from single-cell data to power the prediction of influenza vaccine response

**DOI:** 10.3389/fcimb.2024.1243586

**Published:** 2024-02-07

**Authors:** Xiangyu Ye, Sheng Yang, Junlan Tu, Lei Xu, Yifan Wang, Hongbo Chen, Rongbin Yu, Peng Huang

**Affiliations:** ^1^ Department of Epidemiology, Center for Global Health, School of Public Health, Nanjing Medical University, Nanjing, China; ^2^ Department of Biostatistics, National Vaccine Innovation Platform, Center for Global Health, School of Public Health, Nanjing Medical University, Nanjing, China; ^3^ Department of Infectious Disease, Jurong Hospital Affiliated to Jiangsu University, Jurong, China; ^4^ Department of Epidemiology, National Vaccine Innovation Platform, Center for Global Health, School of Public Health, Nanjing Medical University, Nanjing, China

**Keywords:** influenza, vaccine response, gene expression, single-cell, predictive model

## Abstract

**Introduction:**

Vaccination is still the primary means for preventing influenza virus infection, but the protective effects vary greatly among individuals. Identifying individuals at risk of low response to influenza vaccination is important. This study aimed to explore improved strategies for constructing predictive models of influenza vaccine response using gene expression data.

**Methods:**

We first used gene expression and immune response data from the Immune Signatures Data Resource (IS2) to define influenza vaccine response-related transcriptional expression and alteration features at different time points across vaccination via differential expression analysis. Then, we mapped these features to single-cell resolution using additional published single-cell data to investigate the possible mechanism. Finally, we explored the potential of these identified transcriptional features in predicting influenza vaccine response. We used several modeling strategies and also attempted to leverage the information from single-cell RNA sequencing (scRNA-seq) data to optimize the predictive models.

**Results:**

The results showed that models based on genes showing differential expression (DEGs) or fold change (DFGs) at day 7 post-vaccination performed the best in internal validation, while models based on DFGs had a better performance in external validation than those based on DEGs. In addition, incorporating baseline predictors could improve the performance of models based on days 1–3, while the model based on the expression profile of plasma cells deconvoluted from the model that used DEGs at day 7 as predictors showed an improved performance in external validation.

**Conclusion:**

Our study emphasizes the value of using combination modeling strategy and leveraging information from single-cell levels in constructing influenza vaccine response predictive models.

## 1 Introduction

Influenza infection is a major global public health concern, which causes approximately 3–5 million severe influenza cases worldwide each year and 250,000 to 600,000 deaths (Iuliano et al., 2018). Vaccination is still the most widely used means of preventing and controlling influenza virus infection, but the effectiveness is far from expected (Uyeki et al., 2022). Rapid antigenic evolution of the virus hemagglutinin (HA) and neuraminidase may partly account for the low effectiveness (Uyeki et al., 2022). However, even in seasons with antigenic match between vaccine strains and circulating influenza viruses, vaccine effectiveness reported in outpatients was only 33% to 61% for different circulating strains (Uyeki, 2017). Although various types of influenza vaccines with different dosages, production methods, and inoculation methods have been developed or are currently under development, they are usually applied in special populations and the protective effects still vary widely across individuals (Yamayoshi and Kawaoka, 2019; Teljeur et al., 2022; Uyeki et al., 2022). Therefore, exploring factors that drive differential vaccination responses and identifying individuals at risk of vaccination low response may help improve the protective effects and reduce influenza infection burden from the perspective of precision medicine.

Utilizing systems vaccinology approaches, previous studies have demonstrated the central role of an individual’s immune status in influenza vaccine response, and a number of associations have been identified, especially between gene expression signatures of peripheral immune cells and vaccine response (Tsang et al., 2014; Nakaya et al., 2015; Ovsyannikova et al., 2016; Team et al., 2017). However, most of these studies focused on either expression signatures measured at baseline or early post-vaccination, which represent the pre-existing immune status and that have undergone vaccine stimulation, respectively (Tsang et al., 2014; Nakaya et al., 2015; Ovsyannikova et al., 2016; Team et al., 2017). Although some studies have achieved fairly decent predictive performance using these identified expression predictors, they did not combine the information from baseline and post-vaccination together (Tsang et al., 2014; Team et al., 2017; Avey et al., 2020; Hagan et al., 2022). Recently, several studies also further linked early influenza vaccination-driven transcriptional fold changes (FCs) compared to baseline with later vaccine response, but they instead ignored the influence of pre-existing immunity unexpectedly, which has been proved to have a determinant impact on individuals’ response to influenza vaccination (Avey et al., 2020; Chou et al., 2022; Hagan et al., 2022). In addition, the role of heterogeneity in immune cell composition and function in vaccine responses has been receiving increasing attention (Sparks et al., 2023; Wang et al., 2023), which further offers better opportunities to resolve the immune response to influenza vaccine at single-cell resolution. However, limited attempts have been made to explore its potential in predicting influenza vaccination response.

**Figure 1:**
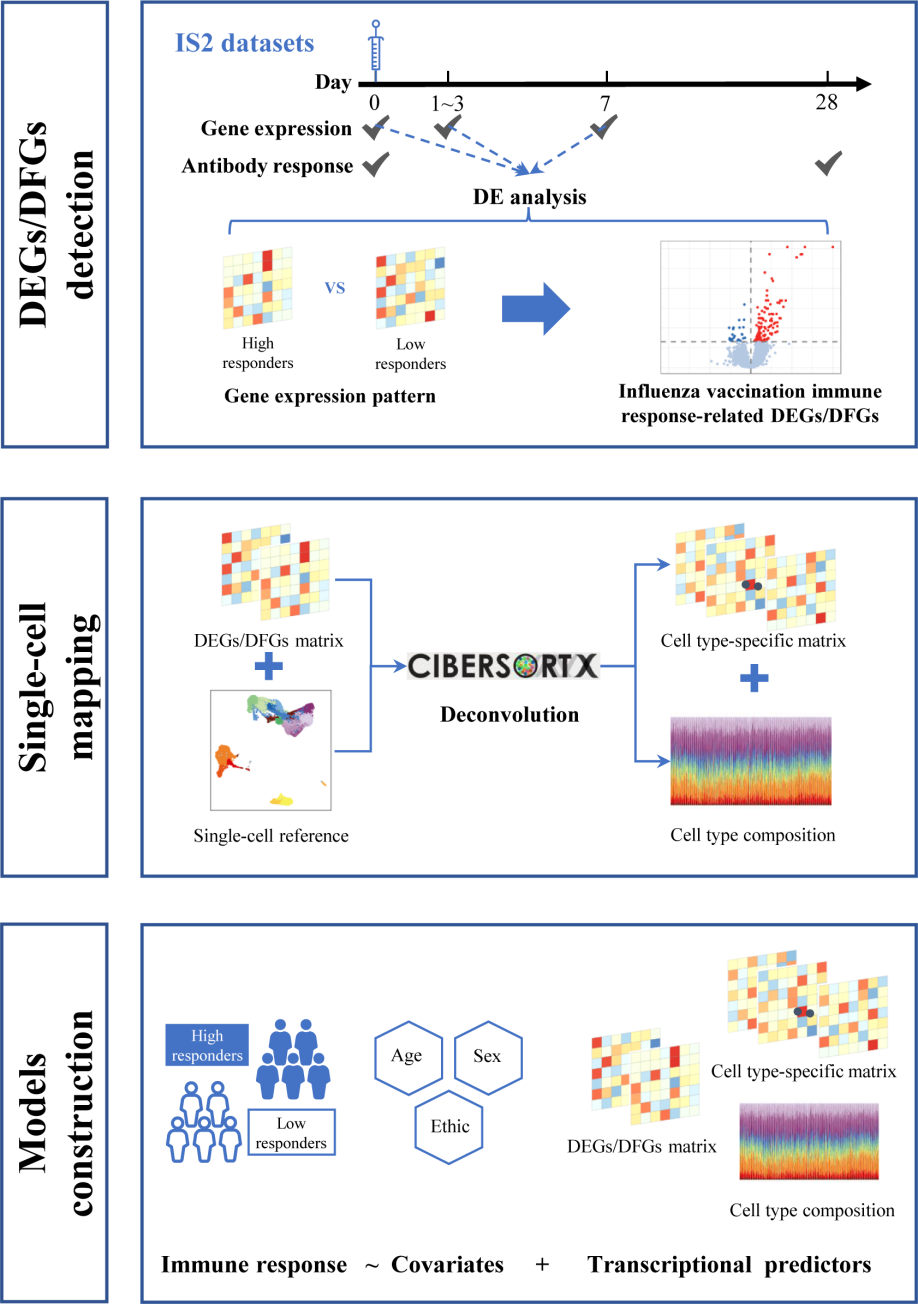
Flowchart for data analysis.

Here, we first integrated gene expression and corresponding immune response data to define influenza vaccine response-related transcriptional features at different time points across vaccination. Then, we mapped these features to single-cell resolution using additional published single-cell data to investigate the possible mechanism. Finally, we treated these identified transcriptional features as predictors and used several modeling strategies to explore their potential in predicting influenza vaccination response. We also attempted to leverage the information from single-cell RNA sequencing (scRNA-seq) data to optimize the predictive models (**Figure 1**).

## 2 Methods

### 2.1 Vaccine immune response data source and preprocessing

Processed microarray or RNA-sequencing (RNA-seq) data and corresponding vaccine immune response data were obtained from the Immune Signatures Data Resource (IS2, https://datatools.immunespace.org/project/HIPC/IS2/begin.view) (Diray-Arce et al., 2022). This dataset includes 1,721 participants from 30 studies, and a detailed description of this dataset and the preprocessing procedure is found in Diray-Arce et al. (2022). In brief, FC of antibody response to vaccine antigen at around 28 days post-vaccination compared to baseline (i.e., day 0 pre-vaccination) was calculated for each participant to evaluate the vaccine immunogenicity (Tsang et al., 2014; Team et al., 2017). Antibody response was evaluated based on neutralizing antibody titers (Nab), hemagglutination inhibition assay (HAI) results, or IgG ELISA assay results (Diray-Arce et al., 2022; Fourati et al., 2022). Following Team et al. (2017), because of multiple strains of viral antigens for the influenza vaccine, we also estimated the maximum FC (MFC) and adjusted MFC (adjMFC) for each participant. Participants were further grouped into three groups: (i) high responders whose MFC value is equal to or above the 60th percentile; (ii) moderate responders whose MFC is below the 60th percentile but above the 40th percentile; and (iii) low responders whose MFC is equal to or below the 40th percentile (Team et al., 2017; Fourati et al., 2022).

### 2.2 Gene expression profile data source and processing

Gene expression data are available on a total of 4,104 samples collected from 1,221 participants, whose immune response data are also documented (Diray-Arce et al., 2022). We downloaded gene expression data containing 10,086 genes that have been cross-study normalized and batch corrected and included samples (i) collected from participants receiving inactivated influenza vaccination; and (ii) collected at baseline, 1–3 days post-vaccination, or 7 days post-vaccination. We also followed Fourati et al. (2022) to exclude samples from moderate responders to minimize the difference in antibody response between studies. Finally, a total of 1,701 samples from 763 participants, namely, 761 samples collected at baseline, 625 samples at 1–3 days post-vaccination, and 315 samples at 7 days post-vaccination, were retained for subsequent analysis (**Supplementary Tables S1, S2**).

Next, we used three procedures to perform RNA-seq data analysis. First, we obtained the expression level for three different time points by making an exponential transformation 2^x^ on the expression matrices. Second, we divided the expression matrices for 1–3 days post-inoculation (FCM_1-3_) and 7 days post-inoculation (FCM_7_) with those of baseline to create FC matrices. We used the *limma* (v 3.50.3) package to identify genes showing differential expression (DEGs) or FC (DFGs) between high and low influenza vaccine responders. DEGs or DFGs were defined as those with Benjamini and Hochberg (BH) adjusted *p-*values< 0.05 (**Table 1**). In particular, we defined influenza vaccine response-related DEGs as genes who have differential expression between high and low responders, which is calculated on a gene expression matrix, while defined response-related DFGs as genes whose fold change in response to vaccination (i.e., fold change from baseline after vaccination) is different between high and low responders, which is calculated on a gene FC matrix. *ReactomePA* (v.1.38.0) and *clusterProfiler* (v.4.2.2) packages were used for enrichment analysis based on Gene Ontology (GO), Kyoto Encyclopedia of Genes and Genomes (KEGG), and Reactome pathway databases (Yu and He, 2016; Wu et al., 2021). Functional items with BH adjusted *p*-value< 0.05 were defined as significant.

**Table 1:** Definitions of DEGs and DFGs.

Name	Definition	Number of case samples (high responders)	Number of control samples (low responders)	Total number of DEGs/DFGs detected
mDEGs_baseline_	Genes that showed marginally significantly differential expression at baseline (unadjusted *p*-value< 0.01) between high and low responders to influenza vaccination	382	379	63
DEGs_day1–3_	Genes that showed significantly differential expression at days 1–3 post-vaccination (BH adjusted *p*-value< 0.05) between high and low responders to influenza vaccination	313	312	0
DEGs_day7_	Genes that showed significantly differential expression at day 7 post-vaccination (BH adjusted *p*-value< 0.05) between high and low responders to influenza vaccination	164	148	191
DFGs_day 1–3_	Genes that showed significantly differential expression between days 1–3 post-vaccination and baseline (BH adjusted *p*-value< 0.05), and whose fold change from baseline to days 1–3 post-vaccination also significantly differed (BH adjusted *p*-value< 0.05) between high and low responders to influenza vaccination	313	311	208
DFGs_day 7_	Genes that showed significantly differential expression between day 7 post-vaccination and baseline (BH adjusted *p*-value< 0.05), and whose fold change from baseline to day 7 post-vaccination also significantly differed (BH adjusted *p*-value< 0.05) between high and low responders to influenza vaccination	167	146	79

### 2.3 Single-cell RNA-sequencing data source and processing

To account for the transcriptome profile at different time points along with influenza vaccination, two published single-cell RNA-sequencing (scRNA-seq) data were downloaded. The first data contained 18 PBMC samples collected from six individuals immunized with influenza vaccine at three time points, including baseline, day 7 post-vaccination, and day 28 post-vaccination (Wang et al., 2023). We chose 12 samples collected at baseline and day 7 post-vaccination, and a total of 86,962 cells were included for subsequent analysis. The second data included six samples from healthy controls collected at day 1 post-vaccination for subsequent analysis, which contained a total of 30,135 cells (Sparks et al., 2023).

In quality control, we first used the *scDblFinder* (v 1.9.4) package on each sample to remove potential doublets, and then we followed the original study to filter out cells: (1) expressing<1,000 or >25,000 unique molecular identifiers (UMIs); (2) expressing<500 or >5,000 genes; or (3) with mitochondrial gene content > 10% of the total UMI count (Germain et al., 2021; Wang et al., 2023). After filtration, a total of 86,874 cells from 18 samples were merged for downstream dimension reduction and clustering analysis using the *Seurat* (v 4.3.0) package (Hao et al., 2021). In brief, the merged data were first normalized and scaled to make it comparable across cells and genes. The top 2,000 high variable genes were also identified. Then, principal component analysis (PCA) was performed based on the variable genes selected for linear dimension reduction, and the *harmony* (v 0.1.1) package was applied to all 50 PCs identified to remove potential batch effects (Korsunsky et al., 2019). We chose the top 29 adjusted PCs, which could cumulatively explain 90.41% variance across cells, for shared nearest neighbor (SNN) graph-based clustering. The dimension reduction and clustering result was finally visualized by the uniform manifold approximation and projection (UMAP). Subsequently, we followed the original study and used canonical markers to annotate the unsupervised clusters identified (**Supplementary Table S3**) (Hu et al., 2022; Wang et al., 2023). In addition, the *AddModuleScore* function in *Seurat* was used to define the score of a pre-defined gene set on the single-cell level by calculating the average expression levels of genes in this set.

### 2.4 Proportion and gene expression deconvolution on bulk expression data

The CIBERSORTx online platform (https://cibersortx.stanford.edu/) was employed to infer the cellular composition and target genes expression from the bulk expression data (Newman et al., 2019; Steen et al., 2020). In brief, we first followed the CIBERSORTx tutorials to build a signature matrix reference file from the processed scRNA-seq count data above (Steen et al., 2020). Note that we disabled the default gene expression-based filtering function as the scRNA-seq count data. We also formatted the bulk expression data that awaited deconvolution into mixture file according to the requirements. Then, we employed the *Cell Fraction* analysis module to deconvolve the proportions of different cell population in the prepared mixture file with the scRNA-seq signature matrix file as reference (Steen et al., 2020). Finally, we run the *High-Resolution* analysis module to recover the sample-level expression profiles of target genes in different cell types from the mixture file (Steen et al., 2020).

### 2.5 Cell-type-associated DEG detection

To determine the impact of cell-type composition on the association between gene expression and influenza vaccine response, we examined the interaction between the proportions of deconvoluted cell populations and expression of DEGs at different time points. Specifically, for each cell-type proportion and the expression of each DEG detected above, we constructed a generalized linear mixed-effects model (GLMM) as follows:


$$\text{Response}∼\text{Covariates}+\text{Gen}{\text{e}}_{\text{i}}+\text{Proportio}{\text{n}}_{\text{j}}+\text{Gen}{\text{e}}_{\text{i}}:\text{Proportio}{\text{n}}_{\text{j}}+(1|\text{study})$$

where $Gene_{i}:\;Proportion_{j}$ denotes the multiplicative interaction term of expression of gene *i* and proportion of cell type *j*, and (1|*study*) denotes the study-specific random effects. Sex, age, and ethnicity were also included as covariates. For each time point and each cell type, we defined cell-type-associated DEGs as those with a BH adjusted *p*-value< 0.05 for the interaction term (Donovan et al., 2020).

### 2.6 Predictive model construction and validation

To explore the predictive potential of identified DEGs and DFGs on influenza vaccine response, we constructed predictive models with DEGs and/or DFGs at baseline, days 1–3 post-vaccination, and/or day 7 post-vaccination as predictors and influenza vaccination response (adjMFC group) at day 28 post-vaccination as response variable. As no significant gene was defined between high and low responders at baseline, we further defined marginally DEGs as those with *p-*values< 0.01 (mDEGs_baseline_), and included them into predictive model construction.

We mainly proposed three modeling strategies (**Table 2**). In the first assumption, we referred to previous studies to take influenza vaccination response-associated DEGs at different time points as predictors, and built three models (models with DEGs_baseline_, DEGs_day1–3_, and DEGs_day7_ as predictors, respectively). In the second assumption, we introduced early transcriptional alterations driven by influenza vaccination (i.e., DFGs), and built another two models (models with DFGs_day1–3 vs baseline_ and DFGs_day7 vs baseline_ as predictors, respectively). Finally, we integrated two kinds of predictors: (i) DEGs or DFGs in response to vaccination, and (ii) DEGs_baseline_. Four models were built on the combination modeling strategy (**Table 2**). Note that in all modeling strategies, sex, age, and ethnicity were treated as covariates.

**Table 2:** Detailed structure of models constructed.

Models	Predictors ^a^	Number of predictors	Number of training samples	Number of external validation samples
Model 1	Covariates + DEGs_baseline_	66	761	57
Model 2	Covariates + DEGs_day 1–3_	3	625	57
Model 3	Covariates + DEGs_day 7_	191	315	57
Model 4	Covariates + DFCs_day 1–3_	167	624	57
Model 5	Covariates + DFCs_day 7_	47	313	57
Model 6	Covariates + DEGs_baseline_ + DEGs_day 1–3_	66	624	57
Model 7	Covariates + DEGs_baseline_ + DEGs_day 7_	250	313	57
Model 8	Covariates + DEGs_baseline_ + DFCs_day 1–3_	204	624	57
Model 9	Covariates + DEGs_baseline_ + DFCs_day 7_	84	313	57
Model 10	Covariates + DEGs_day 7_ in recovered plasma cells	71	313	57
Model 11	Covariates + DEGs_baseline_ + DFGs_day 1–3_ + recovered cellular composition changes at days 1–3 post-vaccination	246	624	57

a Sex, age, and ethnicity were treated as covariates in all modeling strategies.

DEGs, differentially expressed genes; DFCs, differentially expressed fold changes.

For the model fitting, we employed the regularization elastic net regression to estimate parameters and select features (Zou and Hastie, 2005; Kooperberg et al., 2010; Manor and Segal, 2013; Gamazon et al., 2015). We used the *glmnet* package (v 4.1.6) to fit it (Friedman et al., 2010). Based on internal 10-fold cross-validations, we selected the optimal mixing parameter ranging from 0 to 1 with the minimum squared error (Hastie et al., 2009). For performance evaluation, we considered internal fivefold cross-validation and external validation and used the area under the curve (AUC) to evaluate the prediction performance. *pROC* (v.1.18.0) was used to evaluate the performance of the fitted models by calculating the area under the curve (AUC) (Robin et al., 2011). To make a more comprehensive measurement on the performance and the model predictions, we also reported several metrics, including the area under the precision-recall curve (AUPRC), balanced accuracy, F1 score, Matthews correlation coefficient (MCC), specificity, and sensitivity (Vickery, 1970; Matthews, 1975; Kuhn, 2015; Saito and Rehmsmeier, 2015; Saito and Rehmsmeier, 2016).

In external validation, we downloaded another bulk expression data from GEO with accession number GSE194378 and related immune information from Zenodo with Concept RECID 5935844 (https://doi.org/10.5281/zenodo.7566484), which contained 412 samples from 75 participants collected from day 7 pre-vaccination to day 28 post-vaccination (Sparks et al., 2023). We followed the same procedure as above to process the data and applied cutoffs from the training cohorts to the test cohort for grouping. Finally, a total of 171 samples collected at baseline, day 1 post-vaccination, and day 7 post-vaccination from 57 participants, including 30 low responders and 27 high responders, were included for validation, and AUC was used to evaluate the performance of the fitted models on the external data. All analyses were performed in R (v.4.2.0) (Ihaka and Gentleman, 1996).

### 2.7 Statistical analysis

Continuous variables are presented as median (quartile) and compared using unpaired Wilcoxon test. Categorical variables are presented as count (percentage) and compared using *χ* ^2^ test.

### 2.8 Sensitivity analysis

We also conducted a series of sensitivity analyses to demonstrate the robustness of our results. First, in terms of vaccine response definition, considering the wide application of the concept of seroconversion, which is usually defined as an acute-phase serum titer of<10 with a convalescent-phase titer of >40 or a significant increase (>4-fold) in antibody titers between acute- and convalescent-phase serum samples (Greenberg et al., 2009), we re-define participants with seroconversion as high responders and those without seroconversion as low responders and repeated our main analysis. In brief, for the training cohort, a total of 1,813 samples, including 815 samples collected at baseline, 664 samples at 1–3 days post-vaccination, and 334 samples at 7 days post-vaccination, from 817 participants were included for analysis, among which 352 (43.08%) were high responders (**Supplementary Table S2**). For the external validation cohort, a total of 213 samples collected at baseline, day 1 post-vaccination, and day 7 post-vaccination from 71 participants, namely, 30 low responders and 41 high responders, were included for validation (**Supplementary Table S2**). Second, in terms of DEGs/DFGs definition, we took both *p-*values and abs(log_2_(FC)) into consideration, re-defined DEGs/DFGs as those with unadjusted *p-*values*<* 0.01 and abs(log_2_(FC)) > 0.1, and repeated our main analysis. Third, in terms of predictors selection, we tried to take known gene modules as predictors and constructed a series of interpretable models. In brief, we downloaded two sets of known gene modules: the human hallmark gene sets from MSigDB (v.7.4) and the blood transcriptomic modules (BTMs) from Li et al. (Li et al., 2014; Liberzon et al., 2015). We used *AddModuleScore* to calculate gene module scores for each sample, and used Wilcoxon test to identify modules whose scores showed statistical differences between high and low responders. We then took module scores with marginal significance (i.e., with unadjusted *p-*values*<* 0.01) as predictors to construct models, and repeated our main analysis. Finally, in terms of model fitting, we also employed stepwise regression, random forest, and Support Vector Machines (SVM), and repeated our main analysis (Breiman, 2001; Geurts et al., 2006; Steinwart and Christmann, 2008). In brief, for stepwise regression, we performed variance inflation factor (VIF)-based variable filtering from the full model prior to stepwise regression. We set 10 as the cutoff of VIF. When all VIFs are less than 10, we then applied the stepwise regression for Akaike information criterion (AIC)-based model selection. We used *randomForest* to fit random forest (Breiman, 2001). We set the number of trees to 1,000 for each model and at each fold, and the class with at least 50% of votes was defined as predicted response for each sample. We used *e1071* to fit SVM (Dimitriadou et al., 2009). Models were trained using sigmoid kernel function as we found that it had a better performance in our internal cross-validations.

## 3 Results

### 3.1 The identification of DEG and DFG and their functional enrichment in the bulk RNA-seq data

**Figure 2:**
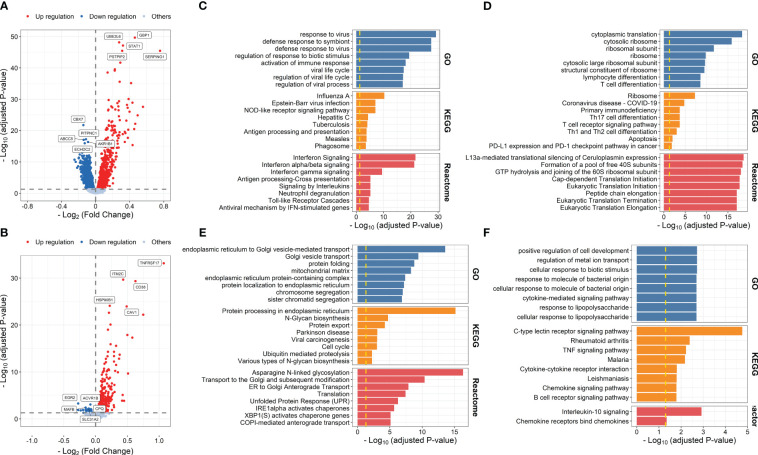
Volcano plots indicating differential expression analysis between samples collected at days 1–3 post-influenza vaccination **(A)** or day 7 post-vaccination **(B)** and those collected at baseline. Bar plots indicating enrichment analysis based on upregulated **(C, E)** or downregulated genes **(D, F)** at days 1–3 post-vaccination **(C, D)** or day 7 post-vaccination **(E, F)** compared with baseline.

We first used the gene expression data and corresponding vaccine immune response data from IS2 to evaluate early transcriptional alterations associated with influenza vaccination. A total of 2,617 DEGs were identified at days 1–3 post-influenza vaccination compared with baseline, among which 1,060 and 1,557 were upregulated and downregulated at days 1–3 post-vaccination, respectively (**Figure 2A**; **Supplementary Table S4**). Gene enrichment analysis based on GO, KEGG, and Reactome pathway databases showed that upregulated genes were mainly enriched in processes, including response to virus (*P* = 5.891 × 10^-30^), defense response to symbiont (*P* = 3.330 × 10^-28^), and regulation of response to biotic stimulus (*P* = 3.857 × 10^-20^), and pathways related to influenza A infection (*P* = 5.084 × 10^-11^), NOD-like receptor signaling pathway (*P* = 9.622 × 10^-8^), and interferon signaling (*P* = 1.922 × 10^-22^), while those downregulated were mainly enriched in immune cell activation and differentiation-related processes and pathways, such as lymphocyte differentiation (*P* = 2.587 × 10^-9^), mononuclear cell differentiation (*P* = 1.232 × 10^-8^), and T-cell receptor signaling pathway (*P* = 5.836 × 10^-6^) (**Figures 2C, D**; **Supplementary Tables S5, S6**). DE analysis between samples from day 7 post-vaccination and baseline defined 720 upregulated and 41 downregulated genes, which were mainly enriched in proteins and nucleic acid processing-related pathways, such as endoplasmic reticulum to Golgi vesicle-mediated transport (*P* = 2.784 × 10^-14^), protein processing in endoplasmic reticulum (*P* = 7.023 × 10^-16^), and asparagine N-linked glycosylation (*P* = 4.745 × 10^-17^), and interleukin signaling and cellular response to molecule-related pathways, such as cellular response to biotic stimulus (*P* = 1.906 × 10^-3^), IL-17 signaling pathway (*P* = 1.665 × 10^-2^), and interleukin-10 signaling (*P* = 1.195 × 10^-3^), respectively (**Figures 2B, E, F**; **Supplementary Tables S7–S9**).

**Figure 3:**
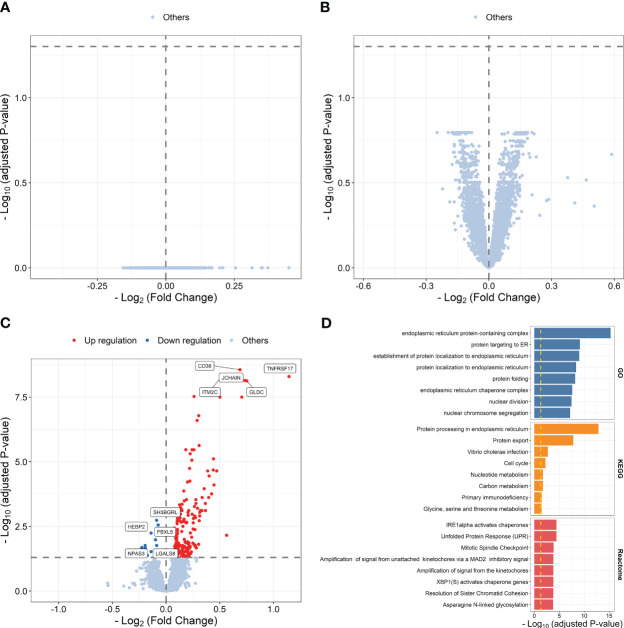
Volcano plots indicating differential expression analysis between high and low responders at baseline **(A)**, days 1–3 post-influenza vaccination **(B)** or day 7 post-vaccination **(C)**. Bar plots indicating enrichment analysis based on upregulated **(D)** genes between high and low responders at day 7 post-vaccination.

We then explored transcriptional differences between high and low responders to influenza vaccine at each time point. The results showed that 191 influenza vaccine response-related DEGs were defined at day 7 post-vaccination, while no response-related DEGs was defined at baseline or days 1–3 post-vaccination (**Figures 3A–C**; **Supplementary Table S10**). Upregulated genes at day 7 post-vaccination were enriched in processes and pathways such as protein targeting to ER (*P* = 7.571 × 10^-10^), protein processing in endoplasmic reticulum (*P* = 1.342 × 10^-13^), and Unfolded Protein Response (UPR, *P* = 3.721 × 10^-5^) (**Figure 3D**; **Supplementary Table S11**).

**Figure 4:**
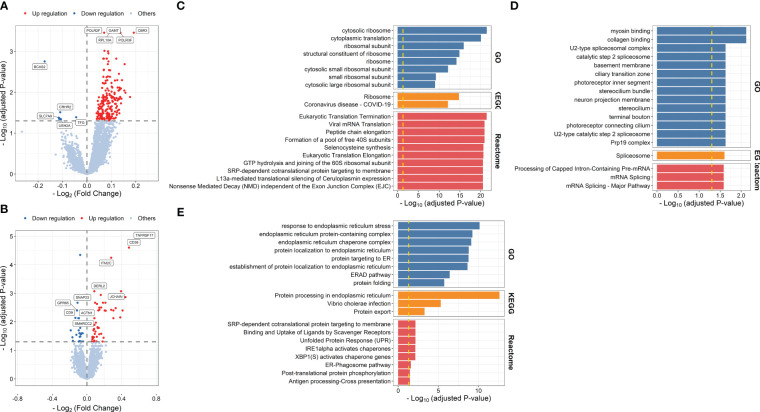
Volcano plots indicating differential expression analysis of FCMs between high and low responders at days 1–3 post-influenza vaccination **(A)** or day 7 post-vaccination **(B)**. Bar plots indicating enrichment analysis based on genes showing relative higher **(C, E)** or lower FCs **(D)** in high responders at days 1–3 post-influenza vaccination **(C, D)** or day 7 post-influenza vaccination **(E)** compared with baseline.

Differential expression analysis identified 208 DFGs at days 1–3 post-vaccination compared with baseline (DFG_day1–3_) (**Figure 4A**; **Supplementary Table S12**). Genes showing relative higher FC in high responders were significantly enriched in processes related to ribosome and pathways related to translation, including ribosome biogenesis (*P* = 2.479 × 10^-7^), rRNA processing (*P* = 1.131 × 10^-6^), and viral mRNA translation (*P* = 1.231 × 10^-21^) (**Figure 4C**; **Supplementary Table S13**), while genes showing relative lower FC in high responders were significantly enriched in processes and pathways related to mRNA splicing, such as catalytic step 2 spliceosome (*P* = 2.351 × 10^-2^) (**Figure 4D**; **Supplementary Table S14**). In addition, 79 DFGs were identified at day 7 post-vaccination compared with baseline (DFG_day7_) (**Figure 4B**; **Supplementary Table S15**), among which those showing higher FC in high responders were mainly enriched in processes and pathways related to endoplasmic reticulum and protein processing, such as response to endoplasmic reticulum stress (*P* = 7.216 × 10^-11^) and protein processing in endoplasmic reticulum (*P* = 2.406 × 10^-13^) (**Figure 4E**; **Supplementary Table S16**).

As we used seroconversion to define vaccine response, we identified much more DEGs and DFGs than those identified based on adjMFC. In particular, we identified a total of 2, 1,966, and 53 influenza vaccine response-related DEGs at baseline (DEGs_baseline_), days 1–3 post-vaccination (DEGs_day1–3_), and day 7 post-vaccination (DEGs_day7_), among which 0, 0, and 43 (81.13%) overlapped with those identified with participants grouped based on adjMFC (**Supplementary Figure S1**; **Supplementary Tables S17, S18**). We also identified 5,419 and 68 DFGs at days 1–3 (DFGs_day1–3_) and day 7 post-vaccination (DFGs_day7_) compared with baseline, among which 201 (3.71%) and 9 (13.24%) overlapped with those on adjMFC (**Supplementary Figure S1**; **Supplementary Tables S19, S20**).

### 3.2 Integration with scRNA-seq data

We mapped our findings to single-cell resolution. A total of 86,874 cells from 18 samples were included for analysis. A total of 21 cell types were defined using cannon marker genes (**Supplementary Figure S2A**; **Supplementary Table S3**). All cell types had a distribution in each sample, which may exclude the presence of an obvious batch effect (**Supplementary Figure S2B**). The results showed that myeloid cells, including CD14+ monocytes, CD16+ monocytes, cDCs, and pDCs, relatively expanded at 1–3 days post-vaccination, while B cells and plasma expanded at 7 days post-vaccination (**Supplementary Figures S2C, D**), which is consistent with sequential activation of innate and adaptive immune responses along with vaccination demonstrated by previous studies (Tsang et al., 2014; Nakaya et al., 2015). In terms of transcriptional alterations, the most shared DEGs were observed in CD14+ monocytes (4,475) and CD16+ monocytes (2,642) at days 1–3 post-vaccination compared to baseline and day 7 post-vaccination (**Supplementary Figures S3, S4**). Of note, 99.70% (35428/35535) of the shared DEGs identified across all cell types at day 7 post-vaccination were downregulated compared to baseline and days 1–3 post-vaccination, with the greatest number identified in CD4+T memory cells (3808) and CD16+ monocyte (3774), while most of the shared upregulated genes were from proliferative T cells (91.59%, 98/107) (**Supplementary Figures S3, S4**).

**Figure 5:**
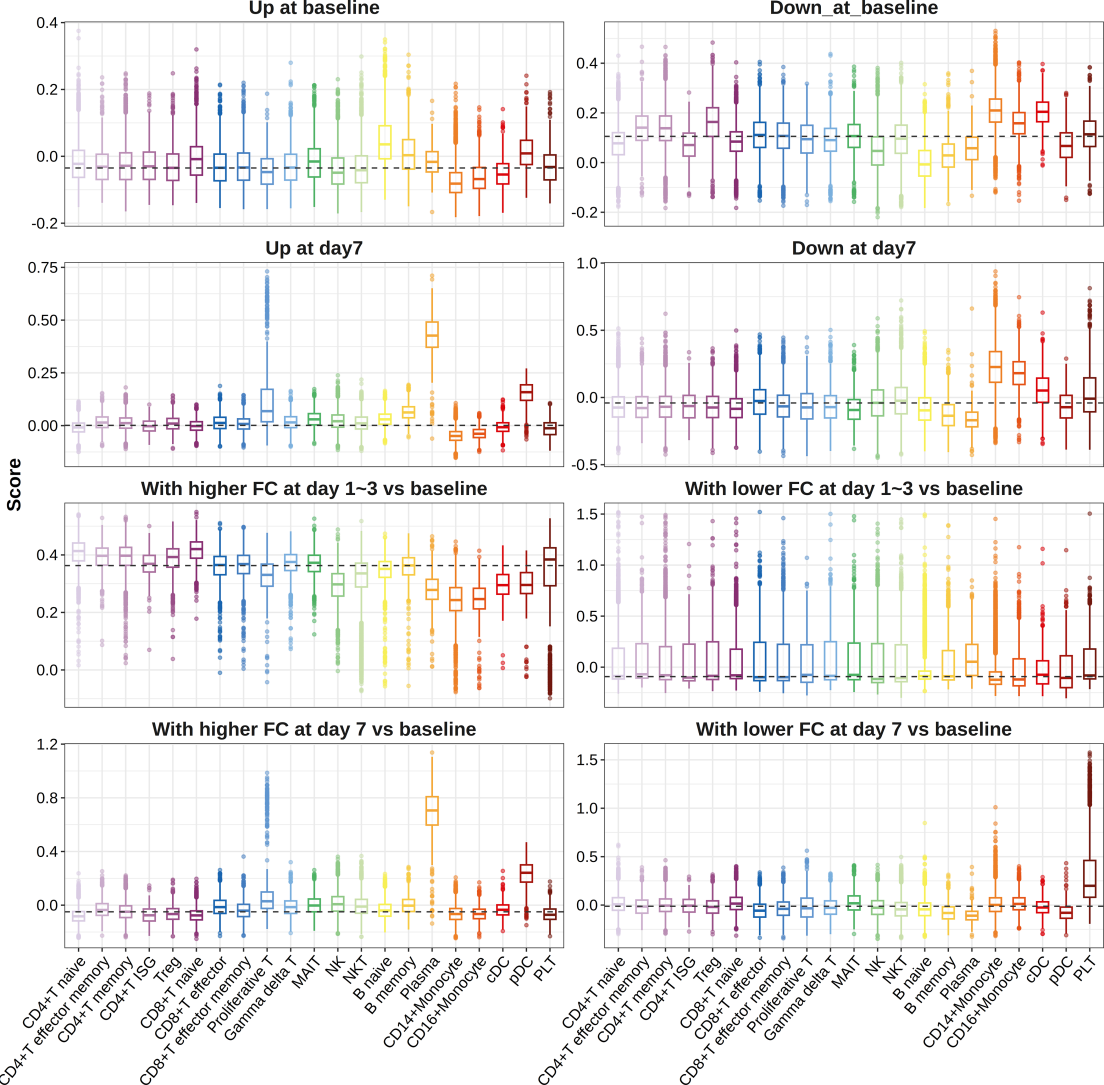
Boxplots indicating expression of gene sets in different cell types. Pre-defined gene sets contained genes upregulated (panels 1 and 3) or downregulated (panels 2 and 4) between high and low responders at baseline (panels 1 and 2), days 1–3 post-vaccination (panels 3 and 4), or day 7 post-vaccination (panels 3 and 4), or those showing relatively higher (panels 5 and 7) or lower FCs (panels 6 and 8) in high responders at days 1–3 post-influenza vaccination (panels 5 and 6) or day 7 post-influenza vaccination (panels 7 and 8) compared with baseline. ISG, interferon stimulated gene; Treg, regulatory T cell; MAIT, mucosal-associated invariant T cell; NK, natural killer cell; NKT, natural killer T cells; DC, dendritic cell; pDC, plasmacytoid dendritic cell; PLT, platelet.

When we mapped influenza vaccine response-associated transcriptional patterns defined above to the single-cell data, we found that genes more highly expressed in high responders at day 7 post-vaccination and those showing higher FC in high responders at day 7 post-vaccination compared with baseline primarily expressed in plasma cells, proliferative T cells, and pDCs (**Figure 5**). However, no cell-type preference was observed for genes showing higher FC in high responders at day 3 post-vaccination compared with baseline (**Figure 5**). A total of 28 cell-type-specific DEGs and 3 cell-type-specific DFGs were identified, among which 27 DEGs and 2 DFGs were defined as plasma cell-specific at day 7 post-vaccination, indicating the great impacts of plasma cell fraction on the gene expression–influenza vaccine response association (**Supplementary Table S21**).

### 3.3 Performance for the prediction models

**Figure 6:**
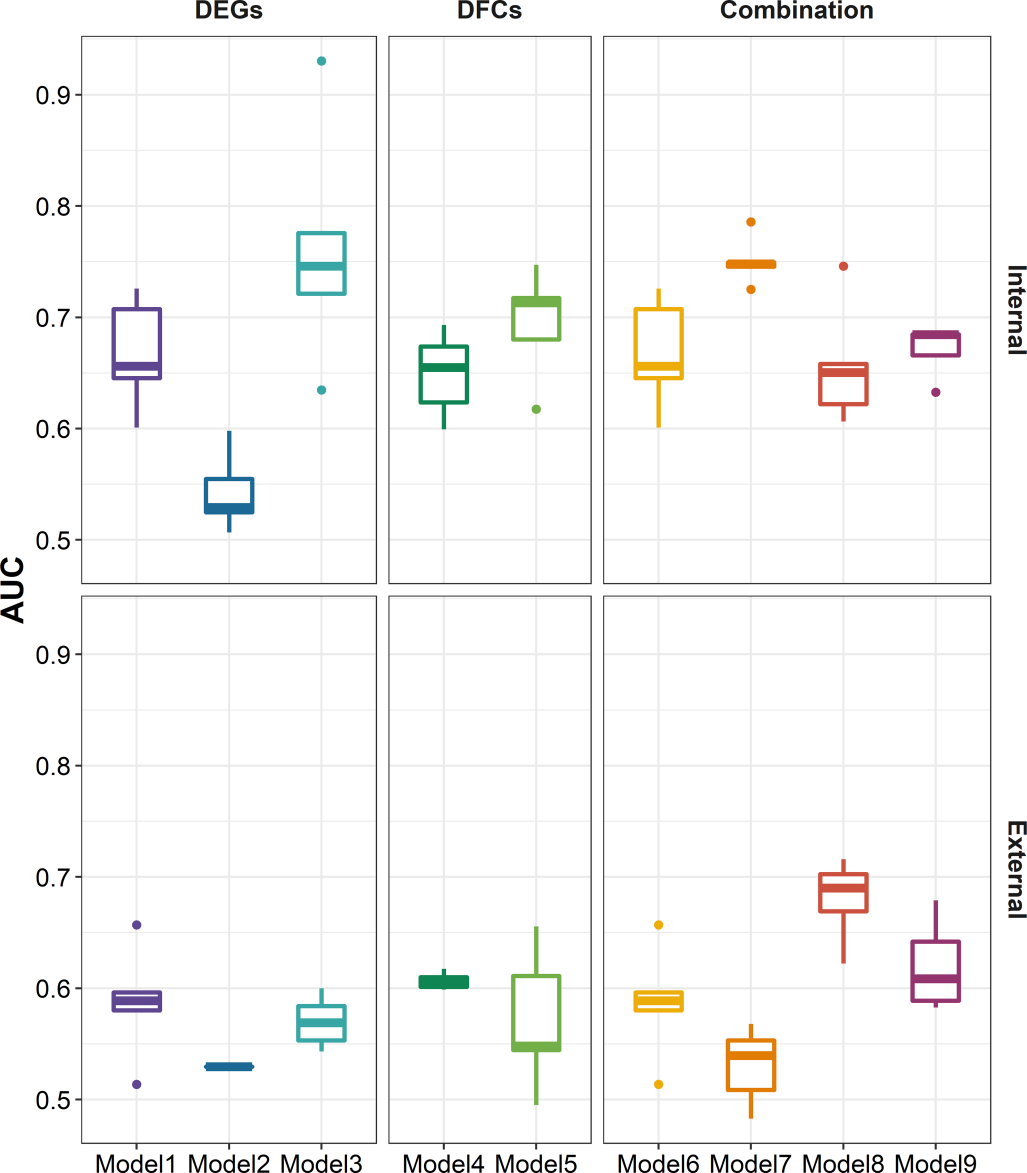
Boxplots indicating AUC of different modeling strategies for predicting response to influenza vaccination using elastic net regression in five-cross internal validation and external validation. Models 1–3 were built using influenza vaccination response-associated DEGs at baseline, days 1–3 post-vaccination, and day 7 post-vaccination as predictors, respectively. Models 4 and 5 were built using influenza vaccination response-associated DFGs at days 1–3 post-vaccination and day 7 post-vaccination as predictors, respectively. Models 6–9 were built on the combination of DEGs at baseline and predictors in models 2–5, respectively. Sex, age, and ethnicity were treated as covariates in all modeling strategies. AUC, area under curve.

After defined transcriptional patterns at baseline or early post-vaccination associated with vaccine response, we next explored their potential in predicting later vaccine response. Taking three main modeling strategies, nine prediction models were constructed (see Methods). In internal fivefold validation, model 3 that used DEGs at day 7 as predictors performed the best with a mean AUC of 0.762, followed by model 7 (mean AUC = 0.751) that used a combination of DEGs at baseline and day 7 as predictors (**Figure 6**). However, in the external validation model, almost all models showed a diminished performance (*P* = 8.379 × 10^-8^, **Supplementary Figure S5**), among which model 7 and model 3 performed the worst, while model 8 that used a combination of DFG_day1–3_ and DEGs at baseline (mean AUC = 0.680), and model 3 that used DFG_day1–3_ only as predictors (mean AUC = 0.620) performed the best (**Figure 6**). Of note, combination strategy could improve the performance of models based on days 1–3 (*P* = 2.773 × 10^-3^), but not for those based on day 7 (**Supplementary Figure S5**). We obtained largely similar results in models fitted using stepwise logistic regression, random forest, and SVM in internal validation (**Supplementary Figures S6–S9**; **Supplementary Table S22**). However, elastic net showed relative better performance in external validation (*P* = 5.155 × 10^-4^ and 9.419 × 10^-7^ for random forest and SVM, respectively), followed by stepwise logistic regression (*P* = 2.121 × 10^-2^ and 8.430 × 10^-4^ for random forest and SVM, respectively), indicating that simple linear combination might be somewhat acceptable for gene expression to predicting vaccination response. We also observed similar results in sensitive analysis that used both *p-*values and abs(log_2_(FC)) to select predictors (**Supplementary Figures S10–S12**). When we used seroconversion to define influenza vaccine response, we observed a significantly higher AUC in internal validation, but lower AUC in external validation (**Supplementary Figures S12–S14**). However, we are surprised to find that models built on selected known gene modules had a relatively poor performance in internal validation, despite comparable performance in external validation (**Supplementary Figures S12, S15, S16**).

**Figure 7:**
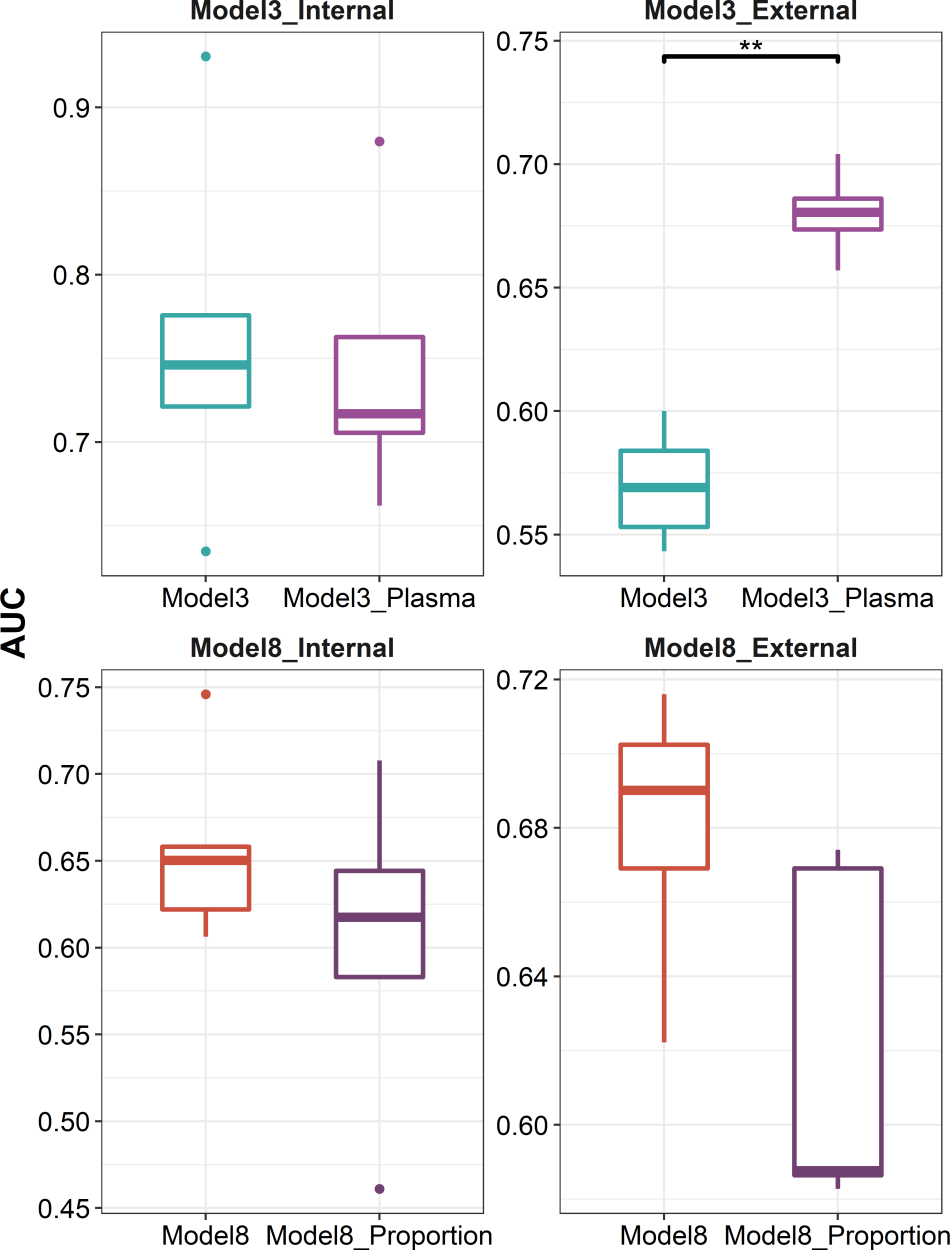
Boxplots indicating comparison of AUC using elastic net regression for models using influenza vaccination response-associated DEGs at day 7 post-vaccination or plasma cell-specific expression profile recovered from these DEGs as predictors (panels 1 and 2), and for models using a combination of influenza vaccination response-associated DFGs at days 1–3 post-vaccination and influenza vaccination response-associated DEGs at baseline or adding recovered cellular composition changes from baseline to days 1–3 post-vaccination additionally as predictors (panels 3 and 4) in internal validation (panels 1 and 3) or external validation (panels 2 and 4). "**" denotes P value < 0.01.

We further explored whether data at the single-cell level can be leveraged to optimize the predictive models. We used CIBERSORTx to deconvolute the cellular composition and cell-type-specific expression of target genes, and found that the predictive model built on the expression profile of plasma cells recovered from influenza vaccine response-associated patterns identified at day 7 post-vaccination showed a better performance in external validation (*P* = 7.937 × 10^-3^, **Figure 7**). As for those identified at days 1–3 post-vaccination, we tried to incorporate the recovered cellular composition changes into model 8 that used a combination of DFG_day1–3_ and DEGs at baseline. However, these composition changes were filtered out in the parameter and variable selection of the elastic net model. As we set the mixing parameter to 0 to use a ridge regression, we still did not get improved AUCs in internal validation or external validation (**Figure 7**).

## 4 Discussion

In this study, we used the DEGs and DFGs to predict the influenza vaccine response. We tried several new model strategies, including combining DEGs and DFGs from both pre- and post-vaccination, and using features deconvoluted from scRNA-seq data. We also compared these models with those built on DEGs and DFGs from a single time point, and performed a series of sensitive analysis to explore the robustness of these strategies. Our study may provide valuable clues for developing improvement predictive models for influenza vaccine response using transcriptional signatures.

In predictor selection, DE analysis between high and low responders to influenza vaccine revealed that at days 1–3 post-vaccination, there were few DEGs but relatively more DFGs compared with baseline, among which *BCAS2* (*P* = 7.042 × 10^-3^) and *USH2A* (*P* = 3.959 × 10^-2^) represented both top DEGs and DFGs downregulated in high responders. *BCAS2* is a pre-mRNA-splicing factor and was reported to play an important role in alternative mRNA splicing and development of oocyte, spermatogonia, and cancer cells, as well as DNA break repair (Kuo et al., 2015; Liu et al., 2017; Wang et al., 2020; Zhang et al., 2022). *USH2A*, which encoded a protein called usherin, was reported to be mainly involved in retinopathy and hearing loss (Toualbi et al., 2020; Dulla et al., 2021; del Castillo et al., 2022). Genes showing relatively lower FC in high responders were also significantly enriched in processes and pathways related to mRNA splicing. In addition, differential expression analysis day 7 post-vaccination also revealed two top DEGs and DFGs upregulated in high responders. The first was *TNFRSF17*, which was known to encode the B-cell maturation antigen and was associated with the pathogenesis and treatment of multiple myeloma and colon cancer (Shah et al., 2020; Song et al., 2022). The second *ITM2C* also encoded proteins expressed on antibody secreting plasma cells and was involved in multiple myeloma (Trezise et al., 2018; Sarıman et al., 2019). Mapping these features to single-cell data also found that DEGs and DFGs upregulated in high responders at day 7 post-vaccination primarily expressed in plasma cells, underlining the critical roles of plasma cells activation at day 7 post-vaccination in favorable vaccine response later. We also observed that the plasma cell fraction at day 7 post-vaccination had a great impact on the association between DEGs_day7_ and influenza vaccine response, suggesting the need to take the proportion of plasma cell into consideration when using DEGs at day 7 to predict vaccine response.

When we further explored the predictive potential of these features identified, we found that models 3 and 7 based on DEGs and DFGs at day 7 performed the best in internal validation, consistent with studies by Hagan et al. and Avey et al. (Avey et al., 2020; Hagan et al., 2022). Notably, three genes in the plasma cell module (M156.1) used by Hagan et al., namely, *TNFRSF17*, *POU2AF1*, and *PNOC*, were also present in our models 3 and 7 (Hagan et al., 2022). Although almost all models showed a diminished performance in external validation, which was also reported in previous studies (Team et al., 2017; Avey et al., 2020), we found that models based on DFGs showed better performance than those based on DEGs, which was also confirmed in our several sensitivity analyses. Using a multicohort analysis, the HIPC-CHI Signatures Project Team and the HIPC-I Consortium identified a baseline transcriptional signature specific to young individuals predictive of influenza vaccination responses (Team et al., 2017). Several studies also found the predictive potential of baseline predictors (Forst et al., 2022; Wang et al., 2022). However, baseline bulk transcriptional features alone did not show an ideal predictive performance in a subsequent study, as well as in our study (Avey et al., 2020). Instead, we found that incorporating baseline predictors could improve the performance of models based on days 1–3, although this did not apply to those based on day 7. As an alternative, the predictive model based on the expression profile of plasma cells deconvoluted from model 3 that used DEGs at day 7 as predictors showed an improved performance in external validation.

Our study also has several limitations. First, we did not evaluate the performance of models based on the expression FC in some specific cell types from baseline to post-vaccination. Although, thanks to CIBERSORTx, we can estimate the expression profile of target genes in specific cell types from the bulk gene expression profile, as our deconvolution is performed separately at each time point, CIBERSORTx may be able to make good estimations on the expression FC in specific cell types from baseline to post-vaccination only when the target gene has a certain variation across samples at both time points (Newman et al., 2019; Steen et al., 2020). Second, since the abs [log_2_(FC)] identified in this study are relatively small, we used only *p-*values to select predictors in our main analysis, which may be somewhat sample size sensitive. We thus added a sensitive analysis by taking both *p-*values and abs [log2(FC)] into consideration for predictor selection. Third, we constrained all of our analysis in participants who received the inactivated influenza vaccine, which may limit the extrapolation of our results. Finally, the scRNA-seq data and bulk transcriptomic data we used were generated from different sources and platforms. Although CIBERSORTx enables robust deconvolution analysis on complex tissues, independent of expression profiling platform or tissue preservation state, as demonstrated by many previous studies (Bohuslavova et al., 2023; Zhang et al., 2023a; Zhang et al., 2023b), it would still be hard for us to exclude bias caused by the differences in data sources.

In summary, leveraging information from baseline predictors and from the single-cell level in predictive model construction could pave the way for vaccine response prediction.

## Data availability statement

The datasets presented in this study can be found in online repositories. The names of the repository/repositories and accession number(s) can be found in the article/**Supplementary Material**.

## Author contributions

RY and PH designed the study. XY, JT, and LX performed the datasets quality control. XY and SY performed the data analysis. RY, PH, and HC interpreted the analysis results. XY, SY, and JT wrote the draft manuscript. SY, YW, and HC revised the article. All authors approved the final manuscript.
