# Supplementary material for: Leveraging baseline transcriptional features and information from single-cell data to power the prediction of influenza vaccine response
Source: Front Cell Infect Microbiol. 2024 Feb 7;14:1243586. doi: 10.3389/fcimb.2024.1243586 (PMC10879619; doi:10.3389/fcimb.2024.1243586)
Supplement: Supplementary file 1 [file Image_1.pdf]

Supplementary Material

Leveraging baseline transcriptional features and information from single-cell data to power the prediction of influenza vaccine response

Xiangyu Ye[†], Sheng Yang[†], Junlan Tu, Lei Xu, Yifan Wang, Hongbo Chen, Rongbin Yu[*], Peng Huang[*]

[†] These authors contributed equally to this work and share first authorship

*** Correspondence:**

Rongbin Yu

rongbinyu@njmu.edu.cn

Peng Huang

huangpeng@njmu.edu.cn

1. Supplementary Figures

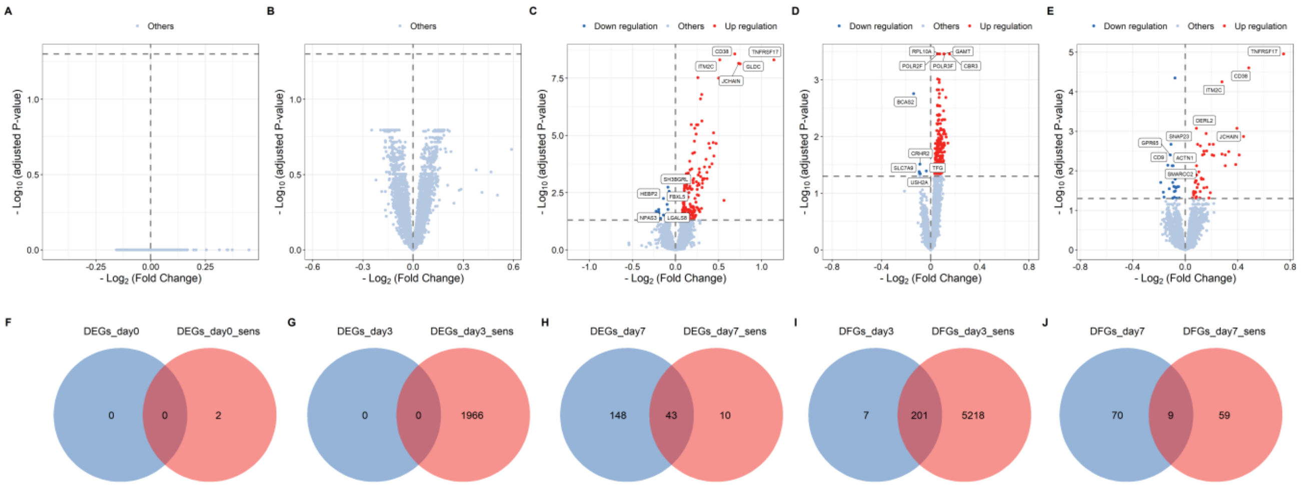

Figure S1 Volcano plots indicating differential expression analysis between high and low responders at baseline (A), day 1~3 post-influenza vaccination (B) or day 7 post-vaccination (C), and differential expression analysis of FCMs between high and low responders at day 1~3 post-influenza vaccination (D) or day 7 post-vaccination (E) using seroconversion to define vaccine response. Venn diagrams indicating the overlap of DEGs (F~H) or DFGs (I, J) with those identified in main analysis that used adjMFC to define vaccine response.

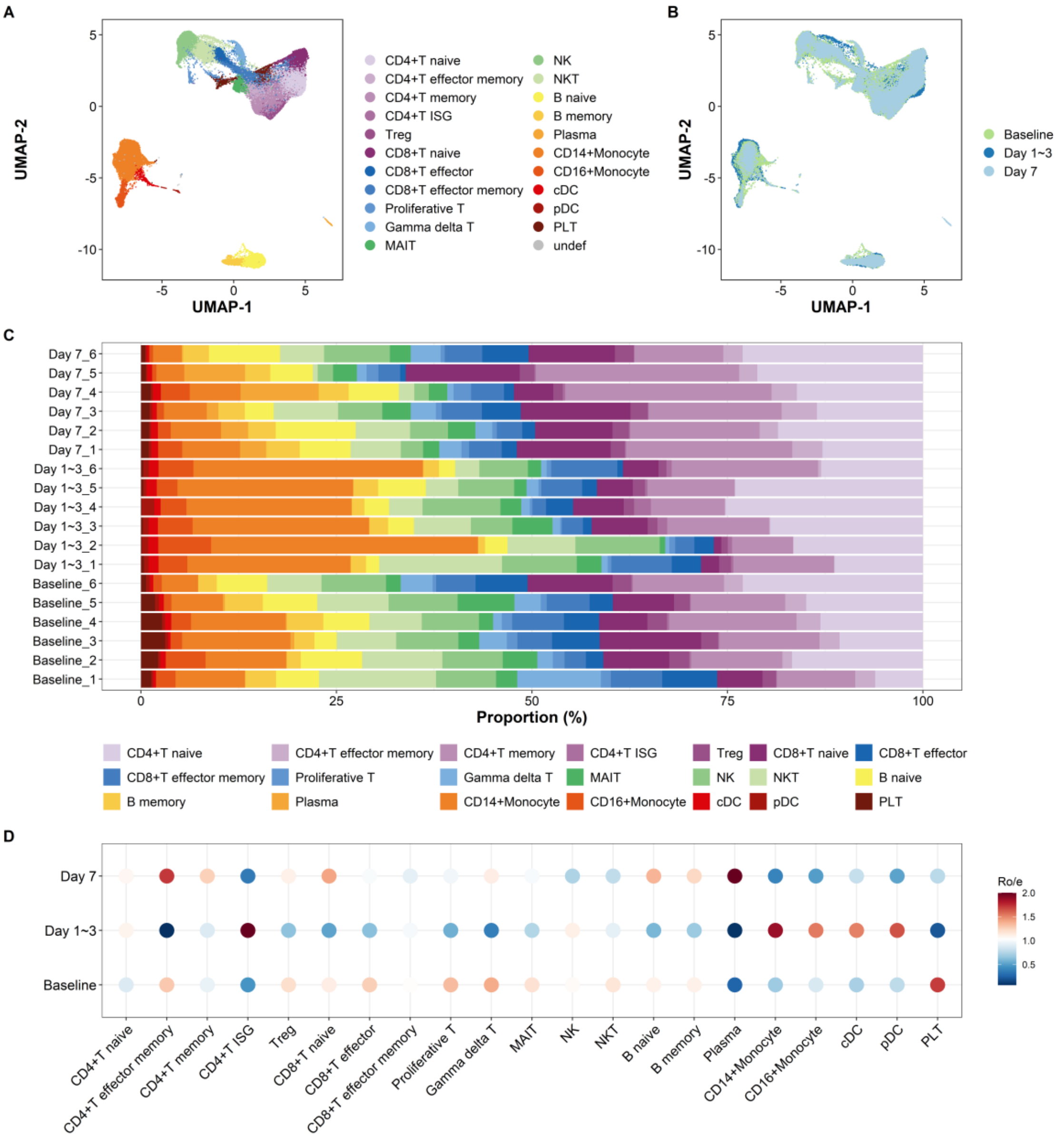

Figure S2 UMAP dimensionality reduction plot for single-cell data colored by cell types (A) and collection time point (B). Bar plot indicating composition of cell types in each sample (C). Bubble plot indicating preferential enrichment of each cell types across three time point (D). Ro/e > 1 indicates enrichment, while Ro/e < 1 indicates depletion. Abbreviation: ISG: interferon stimulated gene, Treg: regulatory T cell, MAIT: mucosal-associated invariant T cell, NK: natural killer cell, NKT: natural killer T cells, DC: dendritic cell, pDC: plasmacytoid dendritic cell, PLT: platelet.

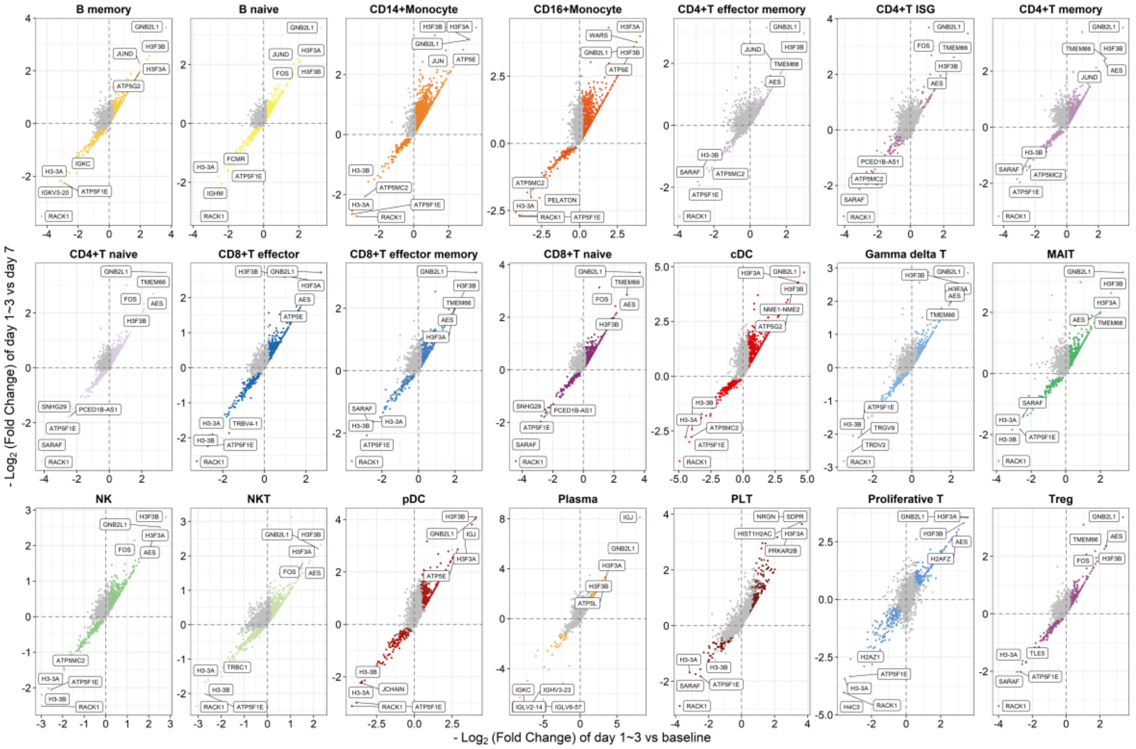

Figure S3 Volcano plots indicating shared differentially expressed genes at day 1~3 post-influenza vaccination compared to baseline (X axis) and day 7 post vaccination (Y axis). Abbreviation: ISG: interferon stimulated gene, Treg: regulatory T cell, MAIT: mucosal-associated invariant T cell, NK: natural killer cell, NKT: natural killer T cells, DC: dendritic cell, pDC: plasmacytoid dendritic cell, PLT: platelet.

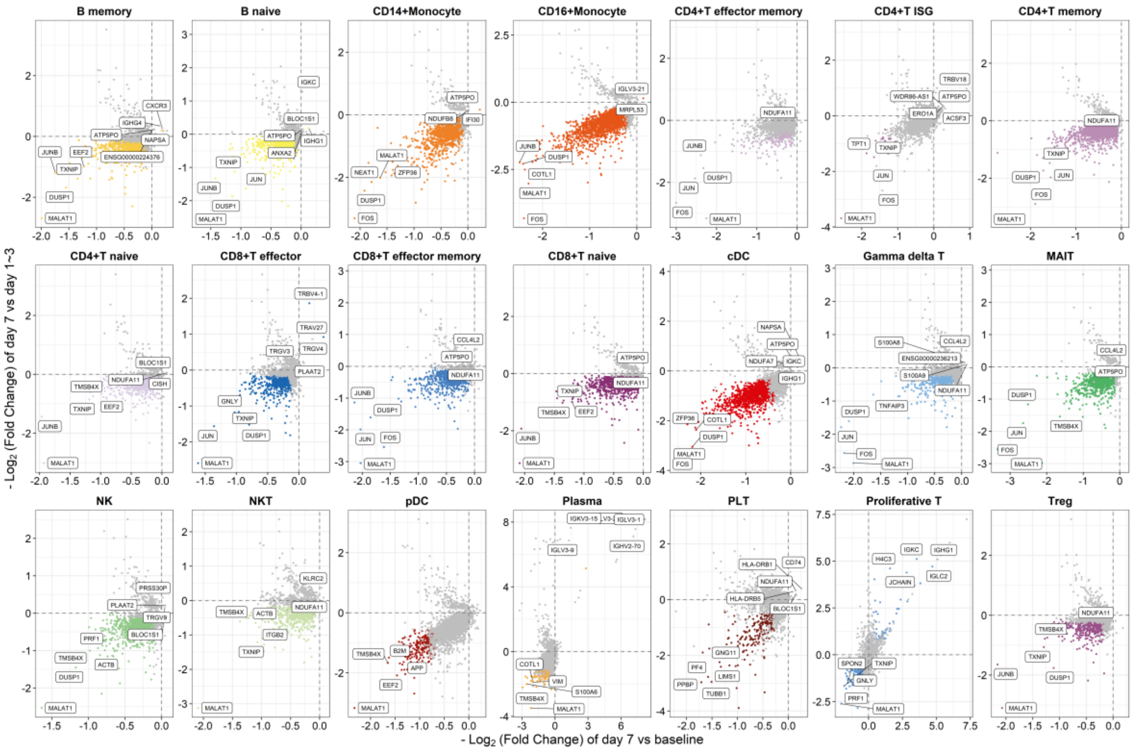

Figure S4 Volcano plots indicating shared differentially expressed genes at day 7 post-influenza vaccination compared to baseline (X axis) and day 1~3 post vaccination (Y axis). Abbreviation: ISG: interferon stimulated gene, Treg: regulatory T cell, MAIT: mucosal-associated invariant T cell, NK: natural killer cell, NKT: natural killer T cells, DC: dendritic cell, pDC: plasmacytoid dendritic cell, PLT: platelet.

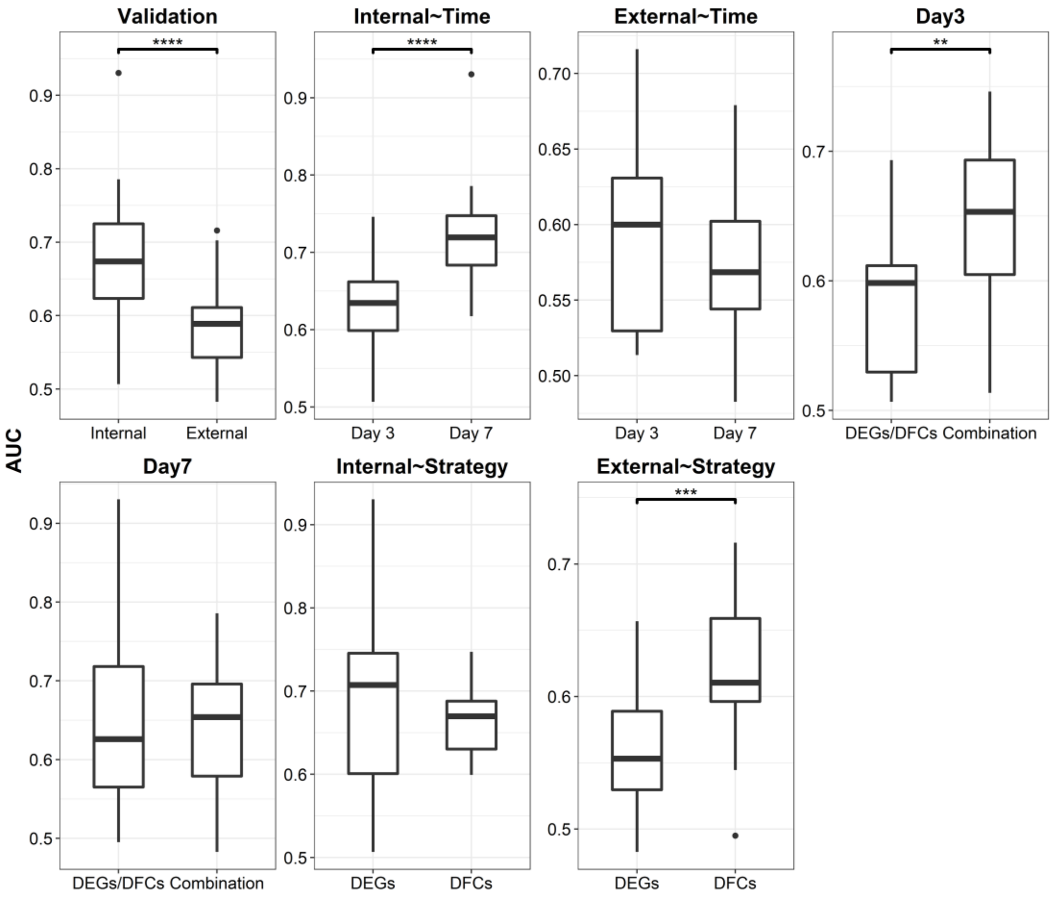

Figure S5 Boxplots indicating comparison of AUCs using elastic net regression for different situations of validation (panel 1), for modeling strategies using predictors from different time points (panel 2 and 3), for modeling strategies without or with a combination with influenza vaccination response-associated DEGs at baseline (panel 4 and 5), and for modeling strategies using DEGs or DFCs (panel 6 and 7). Abbreviation: DEGs: differentially expressed genes, DFCs: differentially expressed fold changes.

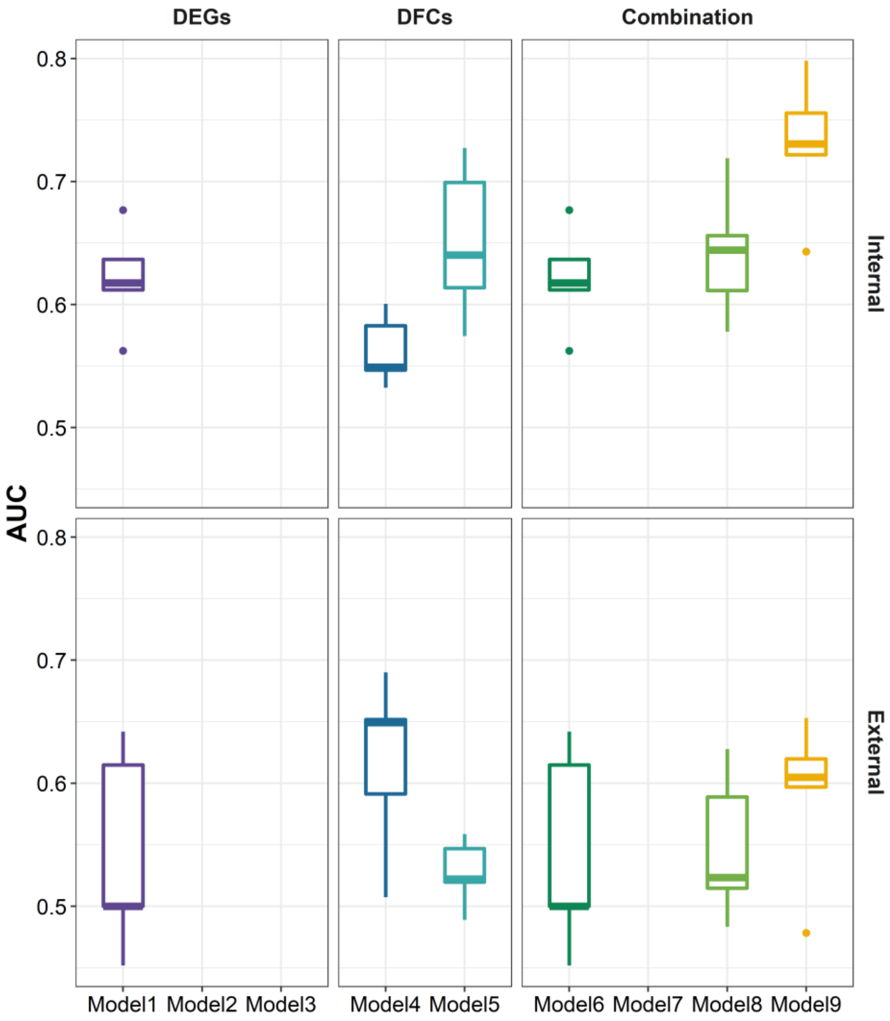

Figure S6 Boxplots indicating AUCs of different modeling strategies for predicting response to influenza vaccination using stepwise regression in five-cross internal validation and external validation. Models 1~3 were built using influenza vaccination response-associated DEGs at baseline, day 1~3 post-vaccination, and day 7 post-vaccination as predictors, respectively. Models 4~5 were built using influenza vaccination response-associated DFCs at day 1~3 post-vaccination and day 7 post-vaccination as predictors, respectively. Models 6~9 were built on the combination of DEGs at baseline and predictors in models 2~5, respectively. Sex, age, and ethnic were treated as covariates in all modeling strategies. Results for model 3 and 7 were not shown because models were fitted with too many predictors but limited observations. Abbreviation: AUC: area under curve.

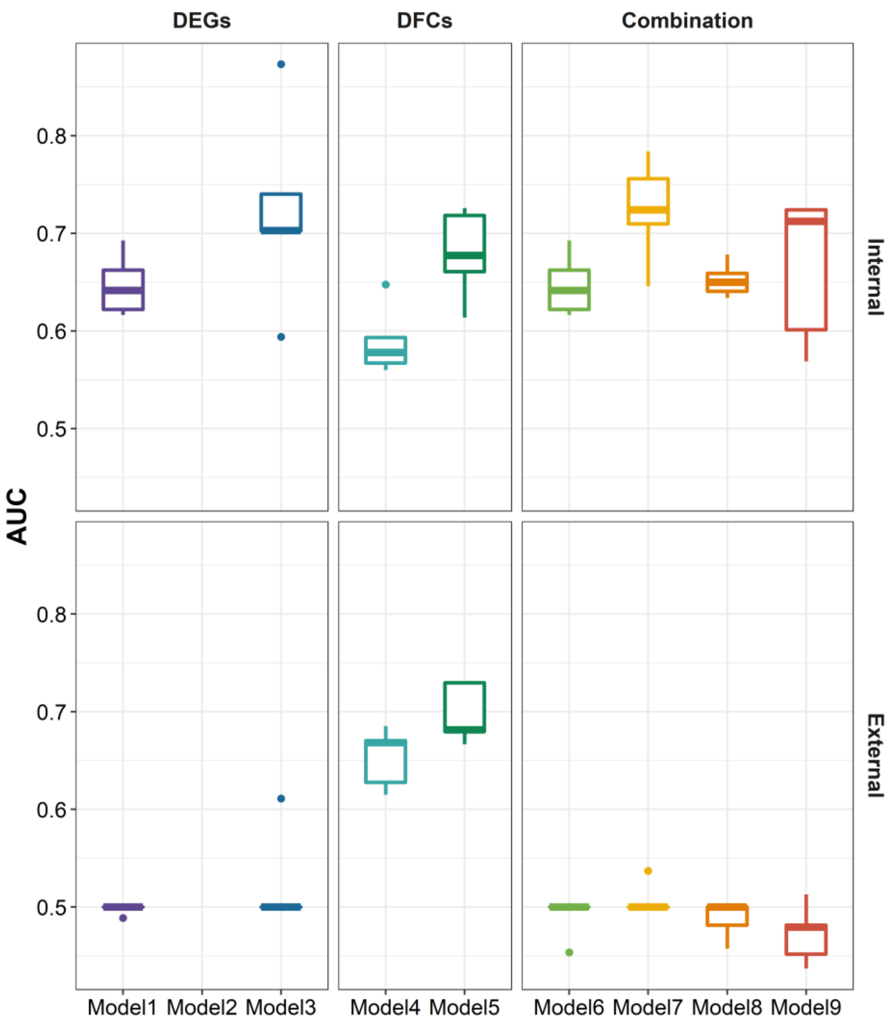

Figure S7 Boxplots indicating AUCs of different modeling strategies for predicting response to influenza vaccination using random forest in five-cross internal validation and external validation. Models 1~3 were built using influenza vaccination response-associated DEGs at baseline, day 1~3 post-vaccination, and day 7 post-vaccination as predictors, respectively. Models 4~5 were built using influenza vaccination response-associated DFCs at day 1~3 post-vaccination and day 7 post-vaccination as predictors, respectively. Models 6~9 were built on the combination of DEGs at baseline and predictors in models 2~5, respectively. Sex, age, and ethnic were treated as covariates in all modeling strategies.

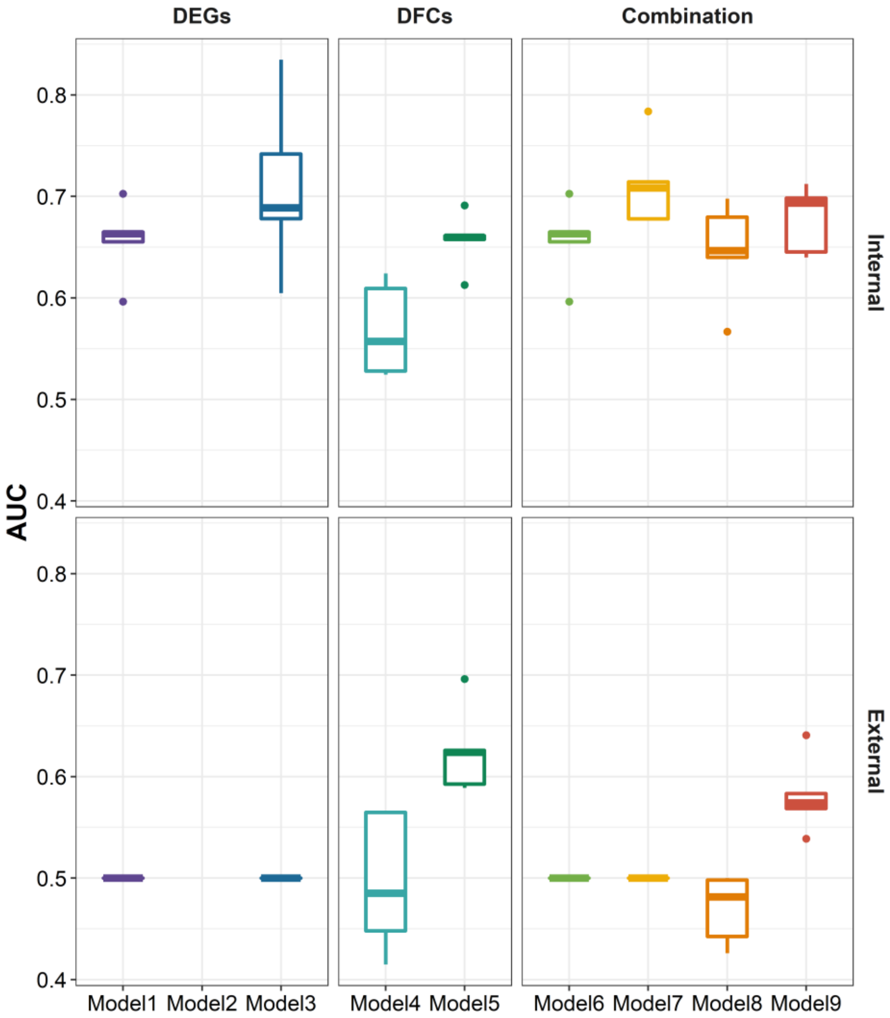

Figure S8 Boxplots indicating AUCs of different modeling strategies for predicting response to influenza vaccination using support vector machine in five-cross internal validation and external validation. Models 1~3 were built using influenza vaccination response-associated DEGs at baseline, day 1~3 post-vaccination, and day 7 post-vaccination as predictors, respectively. Models 4~5 were built using influenza vaccination response-associated DFCs at day 1~3 post-vaccination and day 7 post-vaccination as predictors, respectively. Models 6~9 were built on the combination of DEGs at baseline and predictors in models 2~5, respectively. Sex, age, and ethnic were treated as covariates in all modeling strategies.

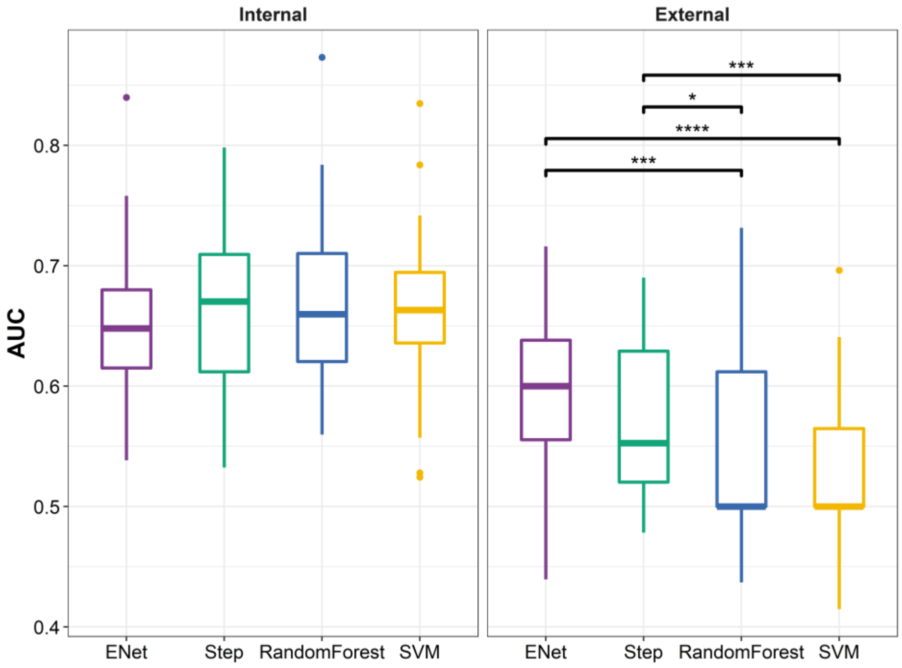

Figure S9 Boxplots indicating comparison of AUCs using elastic net regression and stepwise regression for models in five-cross internal validation and external validation.

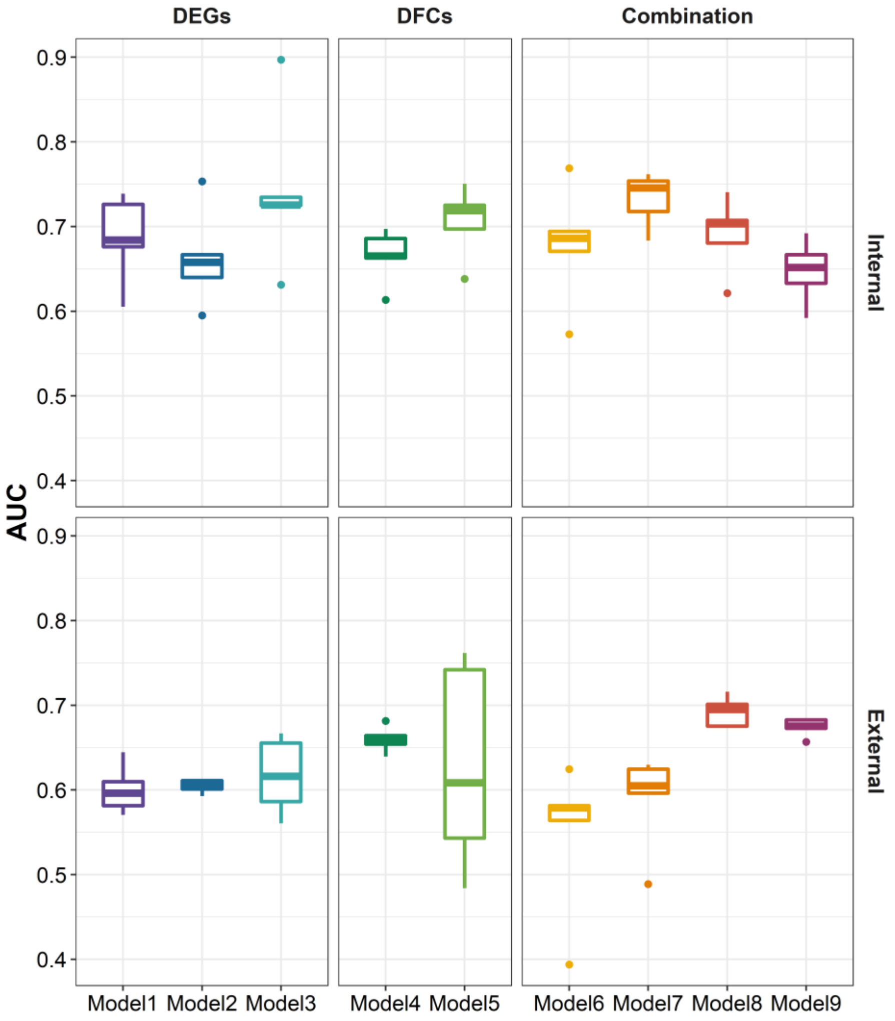

Figure S10 Boxplots indicating AUCs of different modeling strategies for predicting response to influenza vaccination using elastic net regression with predictors defined using both *P values* and abs(log2(FC)) in five-cross internal validation and external validation. Models 1~3 were built using influenza vaccination response-associated DEGs at baseline, day 1~3 post-vaccination, and day 7 post-vaccination as predictors, respectively. Models 4~5 were built using influenza vaccination response-associated DFCs at day 1~3 post-vaccination and day 7 post-vaccination as predictors, respectively. Models 6~9 were built on the combination of DEGs at baseline and predictors in models 2~5, respectively. Sex, age, and ethnic were treated as covariates in all modeling strategies. Results for model 3 and 7 were not shown because models were fitted with too many predictors but limited observations. Abbreviation: AUC: area under curve.

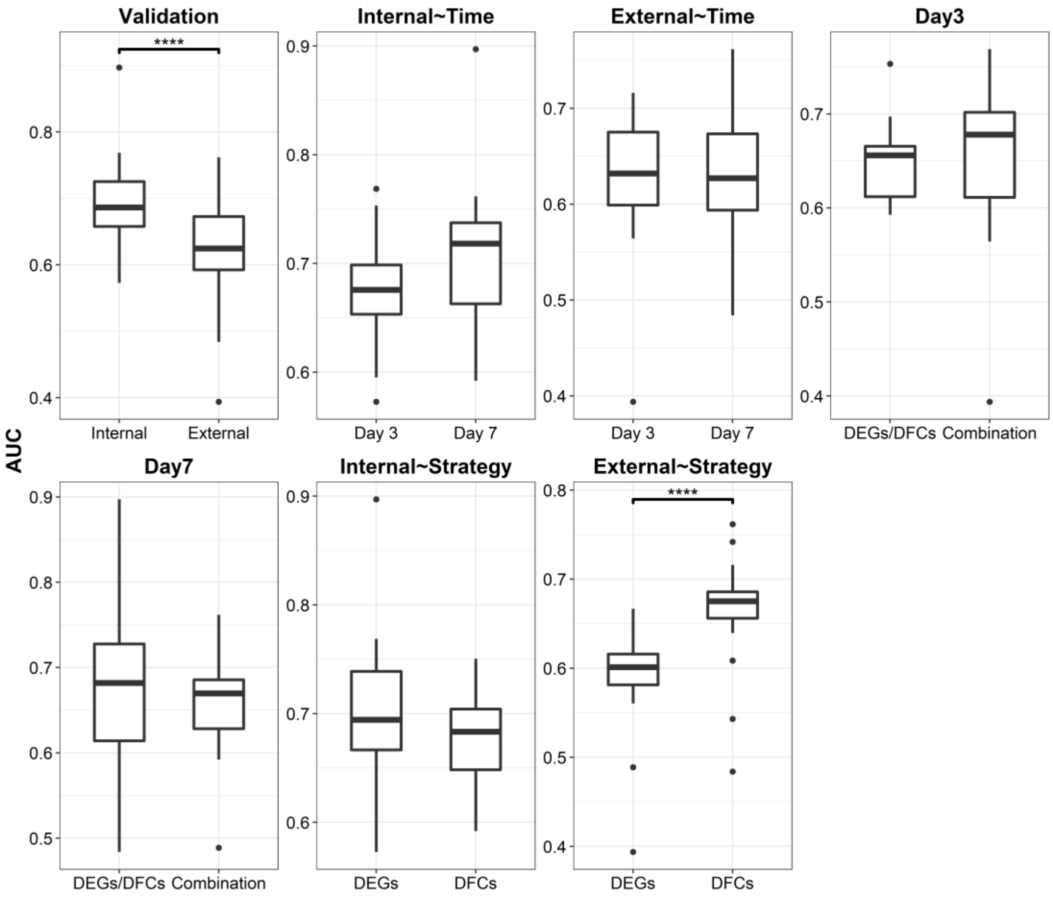

Figure S11 Boxplots indicating comparison of AUC using elastic net regression with predictors defined using both *P values* and abs(log2(FC)) for different situations of validation (panel 1), for modeling strategies using predictors from different time points (panel 2 and 3), for modeling strategies without or with a combination with influenza vaccination response-associated DEGs at baseline (panel 4 and 5), and for modeling strategies using DEGs or DFCs (panel 6 and 7). Abbreviation: DEGs: differentially expressed genes, DFCs: differentially expressed fold changes.

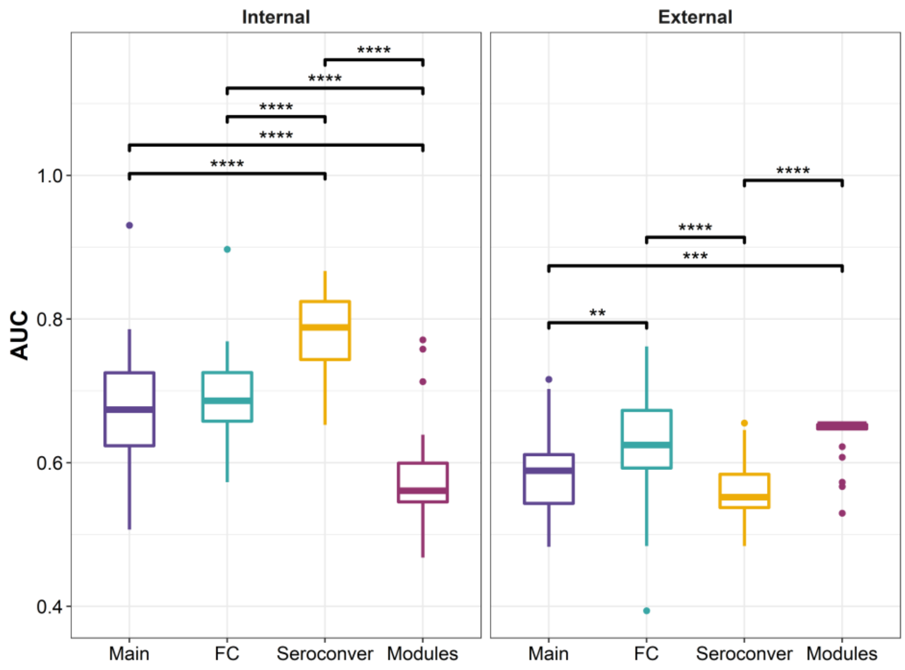

Figure S12 Boxplots indicating comparison of AUC using elastic net regression for models for main analysis and sensitive analysis, including those using predictors defined using both *P values* and abs(log2(FC)), using seroconversion to define vaccine response, and using known gene modules as predictors, in five-cross internal validation and external validation.

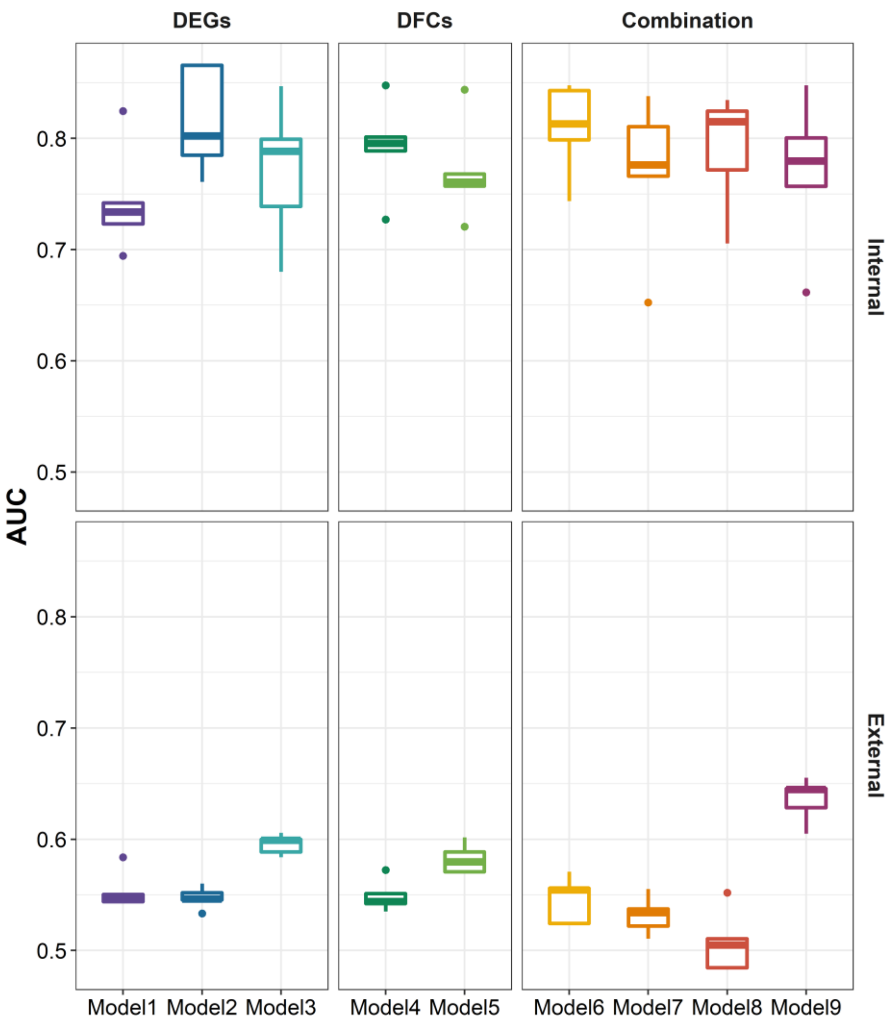

Figure S13 Boxplots indicating AUC of different modeling strategies for predicting response to influenza vaccination using elastic net regression with vaccine response defined by seroconversion in five-cross internal validation and external validation. Models 1~3 were built using influenza vaccination response-associated DEGs at baseline, day 1~3 post-vaccination, and day 7 post-vaccination as predictors, respectively. Models 4~5 were built using influenza vaccination response-associated DFCs at day 1~3 post-vaccination and day 7 post-vaccination as predictors, respectively. Models 6~9 were built on the combination of DEGs at baseline and predictors in models 2~5, respectively. Sex, age, and ethnic were treated as covariates in all modeling strategies. Results for model 3 and 7 were not shown because models were fitted with too many predictors but limited observations. Abbreviation: AUC: area under curve.

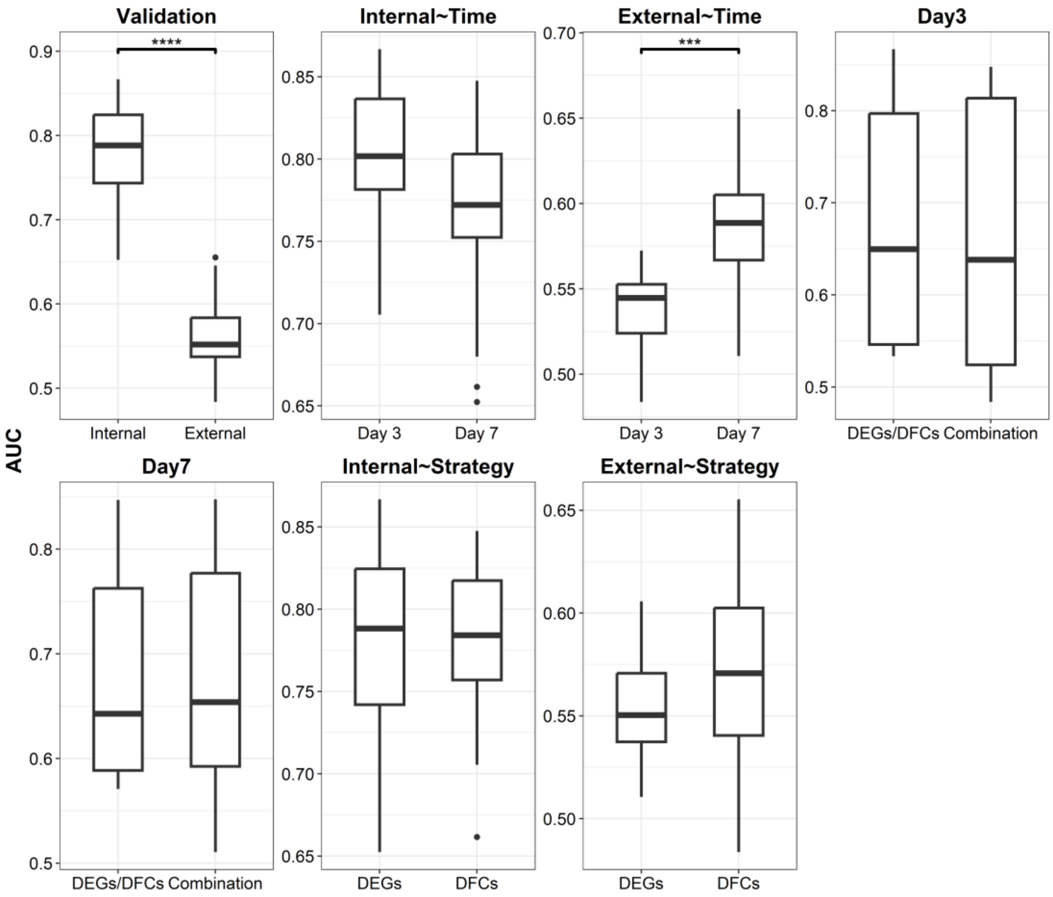

Figure S14 Boxplots indicating comparison of AUC using elastic net regression with vaccine response defined by seroconversion for different situations of validation (panel 1), for modeling strategies using predictors from different time points (panel 2 and 3), for modeling strategies without or with a combination with influenza vaccination response-associated DEGs at baseline (panel 4 and 5), and for modeling strategies using DEGs or DFCs (panel 6 and 7). Abbreviation: DEGs: differentially expressed genes, DFCs: differentially expressed fold changes.

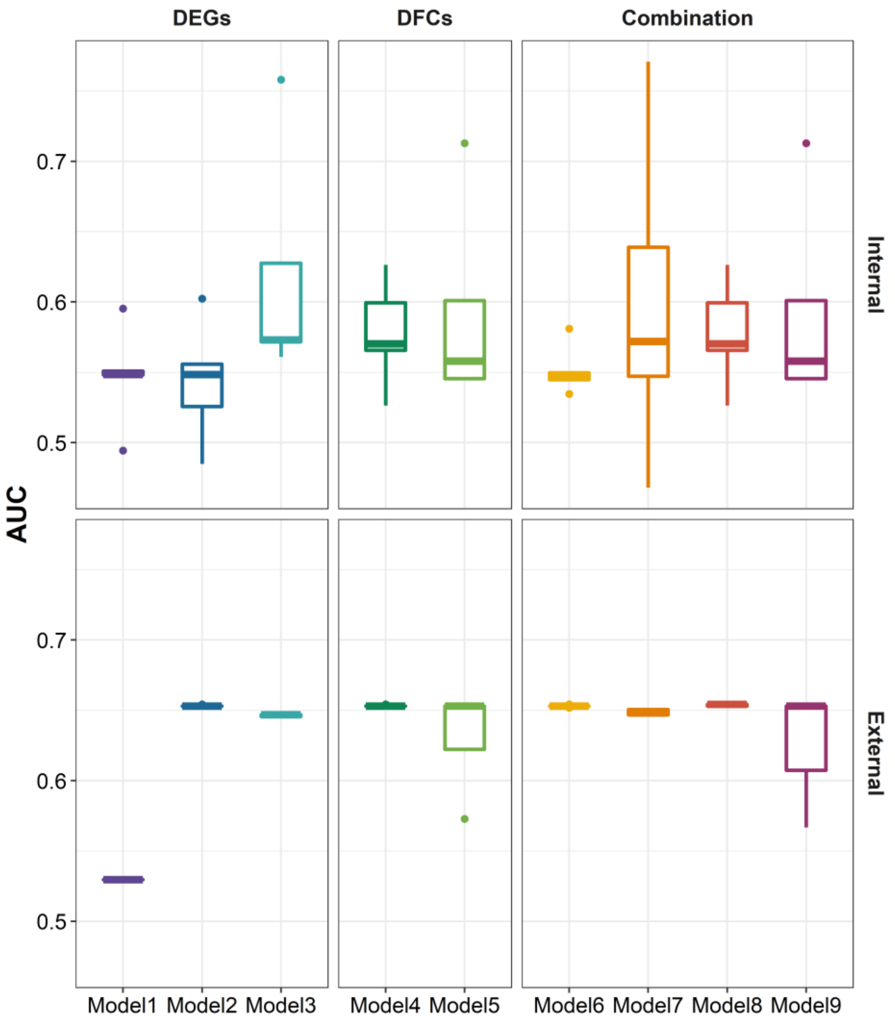

Figure S15 Boxplots indicating AUC of different modeling strategies for predicting response to influenza vaccination using elastic net regression with known gene modules as predictors in five-cross internal validation and external validation. Models 1~3 were built using influenza vaccination response-associated DEGs at baseline, day 1~3 post-vaccination, and day 7 post-vaccination as predictors, respectively. Models 4~5 were built using influenza vaccination response-associated DFCs at day 1~3 post-vaccination and day 7 post-vaccination as predictors, respectively. Models 6~9 were built on the combination of DEGs at baseline and predictors in models 2~5, respectively. Sex, age, and ethnic were treated as covariates in all modeling strategies. Results for model 3 and 7 were not shown because models were fitted with too many predictors but limited observations. Abbreviation: AUC: area under curve.

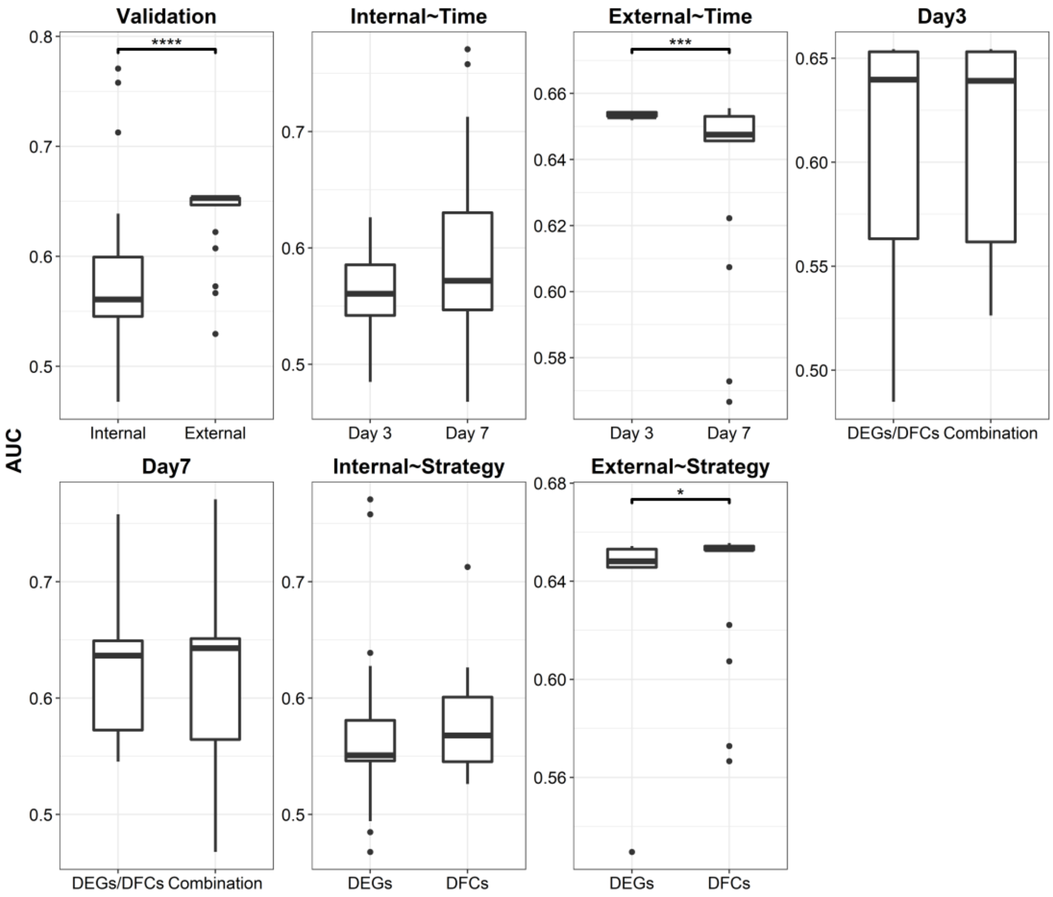

Figure S16 Boxplots indicating comparison of AUC using elastic net regression with known gene modules as predictors for different situations of validation (panel 1), for modeling strategies using predictors from different time points (panel 2 and 3), for modeling strategies without or with a combination with influenza vaccination response-associated DEGs at baseline (panel 4 and 5), and for modeling strategies using DEGs or DFCs (panel 6 and 7). Abbreviation: DEGs: differentially expressed genes, DFCs: differentially expressed fold changes.
